# Revealing the evolutionary history and contemporary population structure of Pacific salmon in the Fraser River through genome resequencing

**DOI:** 10.1093/g3journal/jkae169

**Published:** 2024-07-23

**Authors:** Kris A Christensen, Anne-Marie Flores, Dionne Sakhrani, Carlo A Biagi, Robert H Devlin, Ben J G Sutherland, Ruth E Withler, Eric B Rondeau, Ben F Koop

**Affiliations:** Department of Biology, University of Victoria, Victoria, BC V8W 2Y2, Canada; Department of Biology, University of Victoria, Victoria, BC V8W 2Y2, Canada; Fisheries and Oceans Canada, West Vancouver, BC V7V 1H2, Canada; Fisheries and Oceans Canada, West Vancouver, BC V7V 1H2, Canada; Fisheries and Oceans Canada, West Vancouver, BC V7V 1H2, Canada; Sutherland Bioinformatics, Lantzville, BC V0R 2H0, Canada; Faculty of Science and Technology, Vancouver Island University, Nanaimo, BC V9R 5S5, Canada; Pacific Salmon Foundation, Vancouver, BC V6H 3V9, Canada; Pacific Biological Station, Fisheries and Oceans Canada, Nanaimo, BC V9T 6N7, Canada; Department of Biology, University of Victoria, Victoria, BC V8W 2Y2, Canada

**Keywords:** Chinook, coho, sockeye, genetics, demography, glacial refugia

## Abstract

The Fraser River once supported massive salmon returns. However, over the last century, the largest returns have consistently been less than half of the recorded historical maximum. There is substantial interest from surrounding communities and governments to increase salmon returns for both human use and functional ecosystems. To generate resources for this endeavor, we resequenced genomes of Chinook (*Oncorhynchus tshawytscha*), coho (*Oncorhynchus kisutch*), and sockeye salmon (*Oncorhynchus nerka*) from the Fraser River at moderate coverage (∼16×). A total of 954 resequenced genomes were analyzed, with 681 collected specifically for this study from tissues sampled between 1997 and 2021. An additional 273 were collected from previous studies. At the species level, Chinook salmon appeared to have 1.6–2.1× more SNPs than coho or sockeye salmon, respectively. This difference may be attributable to large historical declines of coho and sockeye salmon. At the population level, 3 Fraser River genetic groups were identified for each species using principal component and admixture analyses. These were consistent with previous research and supports the continued use of these groups in conservation and management efforts. Environmental factors and a migration barrier were identified as major factors influencing the boundaries of these genetic groups. Additionally, 20 potentially adaptive loci were identified among the genetic groups. This information may be valuable in new management and conservation efforts. Furthermore, the resequenced genomes are an important resource for contemporary genomics research on Fraser River salmon and have been made publicly available.

## Introduction

The Fraser River drains a massive and ecologically diverse section of British Columbia, Canada, from the Rocky Mountains to its outlet near Vancouver (Reynoldson *et al.* 2005). The river has at times been the largest producer of salmon in North America (Milne 1964; Northcote and Atagi 1997) and remains important commercially (Henderson and Healey 1993) and culturally for its salmon fisheries. The prodigious declines in salmon, predominantly sockeye and pink salmon from a 1913–1914 landslide caused by the construction of a railway, have never fully recovered (Pacific Salmon Commission—psc.org). There are many pressures potentially for why recovery has not occurred and why there have been continued declines in Fraser River salmon (e.g. fishing, pollution, climate change, and landslides) (Arbeider *et al.* 2020; DFO 2021; Doutaz *et al.* 2023). Understanding these pressures and their influence on salmon will be valuable for governance and conservation.

The Fraser River may have existed in some form for over 66 million years (Tribe 2005). The modern flow of the river is much more recent based on evidence of a major change in flow around 760,000 years ago (Andrews *et al.* 2012). During the last glaciation period (the final in the Pleistocene), the entire region where the modern river is located may have been covered by the Cordilleran ice sheet, which reached its estimated maximum around 19,000 years ago (Clark *et al.* 2009). The retreat of the Cordilleran ice sheet began as early as 18,000 years ago, and there were ice-free regions in British Columbia as early as 17,700 years ago (Warner *et al.* 1982; Darvill *et al.* 2018). Some sections of British Columbia may have even been ice-free during the glacial maximum [e.g. Byun *et al.* (1999), McPhail (2007), and Stewart *et al.* (2009)]. The Cordilleran ice sheet fully retreated around 11,000 years ago, and its only remnants are the glaciers that still exist in modern times (Clague 2017) (see Supplementary Fig. 1 for a map of the estimated glacial recession timeline). These monumental changes in the Fraser River environment likely caused local extinctions of plants and animals from the river. The exact timing of when the Fraser River became accessible again after the glaciers retreated remains uncertain.

If salmon inhabited the Fraser River before the last glacial maximum, they likely went extinct when glaciers covered the river, and colonization or recolonization can be inferred as the source of modern Fraser River salmon. This hypothesis is consistent with the evidence of colonization of other species in this region, such as different plants, deer, and other ray-finned fishes (Soltis *et al.* 1997; Bernatchez and Wilson 1998; Hewitt 2000; McPhail 2007; Latch *et al.* 2009; Beatty and Provan 2010). Colonization or recolonization of the 65–66 species of ray-finned fishes native to British Columbia was thought to have started around 13,000 years ago (McPhail 2007). However, fossil evidence indicates that kokanee, a landlocked ecotype of sockeye salmon (*Oncorhynchus nerka*), could have colonized the interior of British Columbia, possibly via the Columbia River, as early as 18,000 years ago (Harington 1996). This is consistent with a large, isolated and unique kokanee population in the upper Columbia River and Fraser River system that remains today (Beacham and Withler 2017; Christensen *et al.* 2020).

While there is a large body of paleogeographic research and a fossil record describing glaciation and kokanee colonization of the Fraser River, we often do not have information on specific species, locations, or populations. We can turn to genetic studies to complement what we understand from these other 2 fields of study. We expect to observe the genetic legacies of colonization events in modern populations (Hewitt 1996, 2000). Several researchers have suggested that Fraser River salmon have experienced recent genetic bottlenecks related to colonization events (Wehrhahn and Powell 1987; Wood *et al.* 1994; Rondeau *et al.* 2023). These legacies may influence modern populations, and understanding them will be important for evidence-based conservation and management.

Generally, there were 2–3 Fraser River (e.g. lower, middle, and upper) Chinook (*Oncorhynchus tshawytscha*), sockeye, or coho salmon (*Oncorhynchus kisutch*) genetic groups identified from previous studies (Wood *et al.* 1994; Small *et al.* 1998; Teel *et al.* 2000; Beacham *et al.* 2003; Beacham and Withler 2017). This excludes additional groups identified from the Thompson River tributary that were also commonly identified (Small *et al.* 1998; Withler *et al.* 2000; Beacham *et al.* 2003; Beacham and Withler 2017). While these genetic groups could have originated from separate colonization events or different combinations of colonization events, they could also have originated as a result of other factors. These factors include limited gene flow between the groups [e.g. due to barriers like the Fraser Canyon, a phenomenon sometimes referred to as isolation-by-resistance (McRae 2006)], because of isolation-by-distance, adaptation to environmental conditions [sometimes referred to as isolation-by-environment (Weber *et al.* 2017)], ecotype differentiation in sockeye salmon (Beacham and Withler 2017), or through a combination of these mechanisms. If these genetic groups were a result of separate colonization events, it is also possible that the colonizing populations came from different glacial refugia (Wood *et al.* 1994; Small *et al.* 1998; Teel *et al.* 2000; Withler *et al.* 2000), which would increase their overall distinctiveness.

As an example of isolation-by-resistance, Wehrhahn and Powell (1987) note that the Fraser Canyon might limit gene flow between lower Fraser River (LFR) and upper Fraser River (UFR) salmon. Several velocity barriers have been identified in the Fraser Canyon that could influence salmon migration and particularly limit the migration of smaller salmon (Wright 2022). This was dramatically emphasized between 1913 and 1914 when construction left a massive rockslide in the Fraser Canyon that partially blocked some salmon species and completely blocked pink salmon (*Oncorhynchus gorbuscha*) (Kew 1992; Grant and Pestal 2009; Pess *et al.* 2012). This single event reduced salmon returns to around a quarter of previous returns to the Fraser River during peak years (Pacific Salmon Commission—psc.org). If the Fraser Canyon acts as a barrier, we might expect unique genetic characteristics above and below the Fraser Canyon for all the species that must traverse it.

UFR and LFR salmon are also separated by large distances, and because they have natal homing with variable straying [reviewed in Quinn (1993), Keefer and Caudill (2013), and Bett *et al.* (2017)], we expect population structure to be influenced by isolation-by-distance [e.g. Wright (1943), Aguillon *et al.* (2017), and Weber *et al.* (2017)]. In general, we would expect isolation-by-distance to increase the longer that salmon are isolated from each other and with increased distance. We also predict that nearby groups would have higher genetic similarity than those further away for most species. An exception would be salmon with different life history types (e.g. odd and even-year pink salmon (Christensen *et al.* 2021)] as this can cause reproductive isolation. Sockeye salmon and kokanee, in the current study, might also be considered an exception in some instances (Christensen *et al.* 2020).

The expectation of increased genetic distance with increased geographic distance would not be the main driver of differentiation if genetic groups were largely influenced by separate colonization events. If colonization occurred by different genetic groups (e.g. populations from different glacial refugia), we might expect genetic distance to be unrelated to geographic distance without strong gene flow or long periods of time. Patterns of colonization from different genetic groups would also be expected to be variable among species.

Adaptation to the environment is of particular interest for the conservation and management of a species because salmon from an area could be uniquely suited to that region. The headwaters of the Fraser River flow north down the Rocky Mountain Trench, which leads to the Interior Plateau, followed by the Fraser Canyon, and finally the river outlets through a major delta near the metropolis of Vancouver, British Columbia (Reynoldson *et al.* 2005). These regions have variable climates, elevations, and river velocities (Reynoldson *et al.* 2005). They are also separated by great distances. Genetic signatures correlated with environmental factors could be from neutral factors such as isolation-by-distance or nonneutral factors such as local adaptation or other forms of selection. Researchers have not yet found a reliable method for distinguishing among the different mechanisms with genetic data alone (reviewed in Bierne *et al.* (2013), Lotterhos and Whitlock (2015), Ahrens *et al.* (2018), and Saravanan *et al.* (2020)]. Additional experiments are necessary to firmly establish specific genetic adaptations.

This is the first study to use whole-genome resequencing data for a large distribution of multiple Fraser River salmon species. The resolution of resequencing allows us to compare metrics previously unavailable in an unbiased manner (e.g. genetic diversity and effective population size). In this study, we analyze 954 resequenced genomes from 3 species of salmon (317 sockeye, 360 Chinook, and 277 coho salmon), mainly from the Fraser River, to understand differences among the species, to identify genetic groups among the collections of samples within each species, and to characterize genetic adaptation to different environments. Data related to genetic structure and local adaptation are especially relevant to conservation and management. By using data from multiple species, we compare among species to identify common patterns. This work provides a foundational data set that will be openly available and of significant use to the salmonid research community for years.

## Materials and methods

### Sampling and whole-genome resequencing

**Fig. 1.**
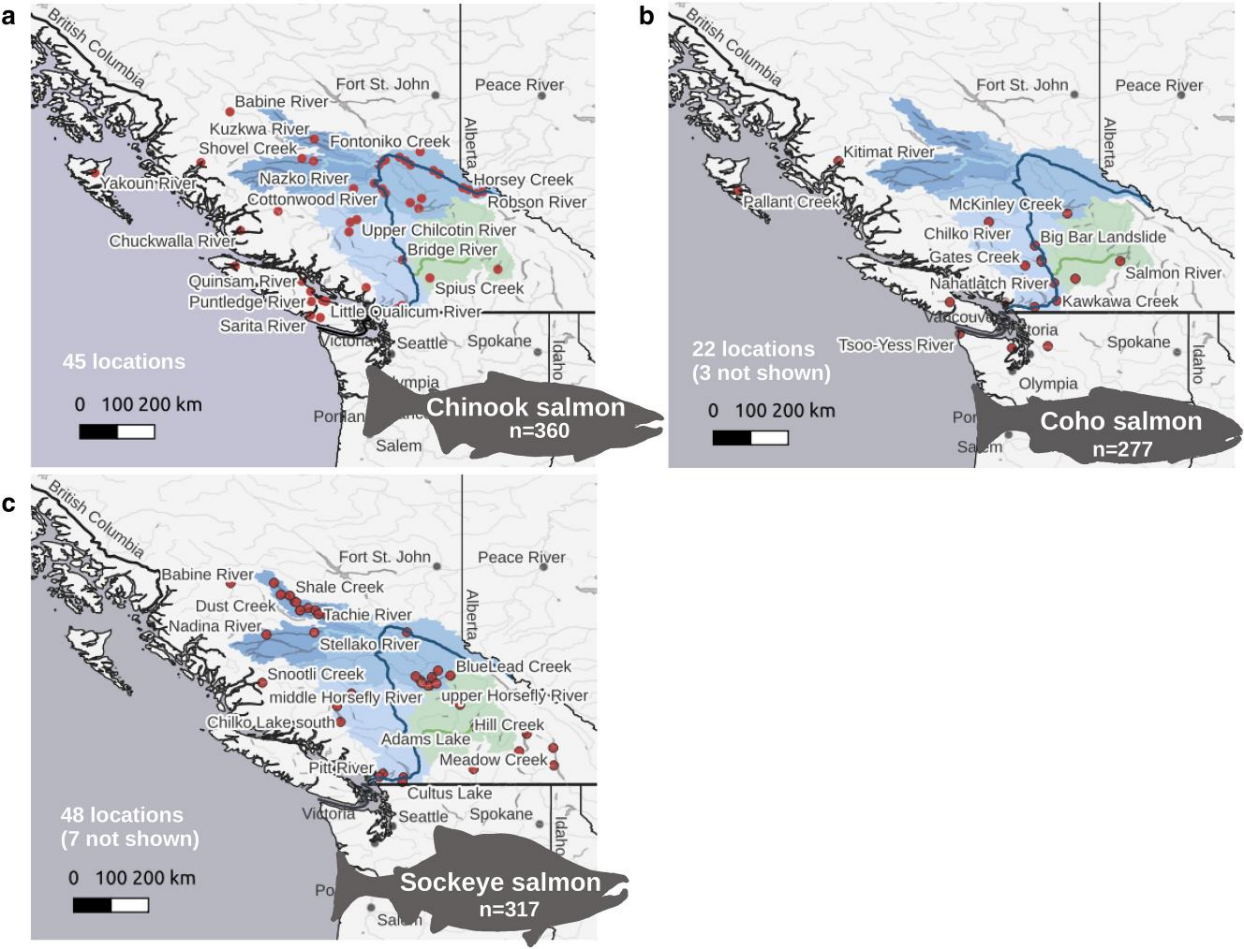
Fraser River salmon sampling locations. Sampling locations of a) Chinook salmon (an average of 7.8 samples per location), b) coho salmon (an average of 6.9 samples per location), or c) sockeye salmon (an average of 6.5 samples per location). Each location is shown as the closest point of the specified body of water to the Fraser River. The Fraser River and associated watersheds are highlighted on the map. The Nechako River is highlighted as the top left watershed (darker blue watershed, with the river as a lighter blue), and the Thompson River is highlighted as the bottom right watershed (green, with the river as a darker green). Watershed and geographical data are from ced.org and naturalearthdata.com, respectively. The map was generated using QGIS software. Not all sampling locations could be displayed on these maps. Information on sampling locations that are outside the boundaries of these maps can be found in Supplementary File 1 and Supplementary Fig. 2.

Chinook, coho, and sockeye salmon were sampled from Fraser River tributaries and lakes, or they were from other bodies of water in previous studies [Fig. 1, Supplementary Fig. 2, Supplementary File 1, (Christensen *et al.* 2020; Rondeau *et al.* 2023)]. Samples from preceding studies that overlapped with bodies of water in the current study were from different individuals. Some of the sampling locations include hatchery sources (Supplementary File 1). Most modern hatchery stocks originate from local sources [e.g. Heard (2012)], and they should reflect the current Fraser River population if not the historical population. The samples were collected between 1997 and 2021 (Supplementary File 1). There were 125 coho salmon collected at the Big Bar Landslide between 2019 and 2021. Spawning locations were unknown for these samples. These were included to identify potentially unknown genetic structure since it is difficult to reach UFR sites during coho salmon spawning.

Samples were collected by Fisheries and Oceans Canada personnel in compliance with the Canadian Council on Animal Care Guidelines and under the authority of the Fisheries and Oceans Canada Pacific Region Animal Care Committee (Ex.7.1). Samples were taken either as operculum clips or as scales. Both were desiccated and stored on Whatman paper.

Genomic DNA was extracted from tissue, after an overnight incubation in 95% ethanol, using a Quick-DNA Kit (Zymo Research). Other genomic DNA samples were previously extracted by Fisheries and Oceans Canada using automated BioSprint extractions, as per the manufacturer's instructions (Qiagen). DNA samples were then sent to the Michael Smith Genome Science Centre (Vancouver, BC) for library preparation and whole-genome resequencing.

Whole-genome sequencing libraries were prepared by shearing the DNA samples individually using a Covaris LE220 (duty cycle: 20%, PIP: 450, cycles per burst: 200, time per run: 90 s; with pulse spin after 45 s). Individual libraries were then constructed using the MGIEasy PCR-Free DNA Library Prep Set (MGI Tech Co.). The indexed libraries were then pooled and sequenced on an MGISeq-G400 sequencer (paired-end 200 bp). Data used in this study were also taken from previous studies (Christensen *et al.* 2020; Rondeau *et al.* 2023) that used Illumina sequencing technology.

### SNP calling and filtering

Reads from each individual were aligned to the reference genome assembly of the respective species [Chinook salmon: GCF_018296145.1 (Christensen *et al.* 2018), coho salmon: GCF_002021735.2 (Rondeau *et al.* 2023), and sockeye salmon: unreleased version 2 (Christensen *et al.* 2020)] with BWA (version 0.7.17, parameter -M) (Li and Durbin 2009, 2010; Li 2013). Reads were sorted with SAMtools (version 1.12, default parameters) (Danecek *et al.* 2021). Picard (version 2.26.3, default parameters) (Broad Institute 2019) was used to add read group information and mark reads that were suspected PCR duplicates. GATK (version 3.8) (McKenna *et al.* 2010; Van der Auwera *et al.* 2013) was then used to call nucleotide variants for each individual (parameters: -T HaplotypeCaller, –genotyping_mode DISCOVERY, –emitRefConfidence GVCF) and then combined (parameters: -T GenotypeGVCFs, –max_alternate_alleles 3). Truth and training SNP data sets (Supplementary File 2) were then used to recalibrate nucleotide variant scores and filter variants using GATK (parameters -T ApplyRecalibration, –mode SNP –ts_filter_level 99.5). The truth SNPs came from multiple studies (Brieuc *et al.* 2014; Meek *et al.* 2016; Nichols *et al.* 2016; Larson *et al.* 2017; Veale and Russello 2017; Rondeau *et al.* 2023), and the training SNPs are described in Supplementary File 2.

Additional filters were used to remove indels, SNPs with more or fewer than 2 alleles, or that were missing genotypes in more than 10% of the individuals. SNPs were removed if their mean depths were outside a range of 8–100× and if they had less than 0.01 minor allele frequency (MAF). All filtering was performed using VCFtools (version 0.1.15) (Danecek *et al.* 2011). The MAF would eliminate alleles found in fewer than 6–8 heterozygous individuals (or 3–4 homozygous individuals), depending on the species and its respective sample size. This threshold was chosen to reduce sequencing errors but to keep all but the rarest variants. The MAF filter was not used for the SMC++ analysis. Linkage disequilibrium (LD) was evaluated and used to filter SNPs in some analyses, as noted below. The prune add-on for BCFtools (version 1.9) (Danecek *et al.* 2021) was used to filter based on LD (parameters: +prune, −w 20 kb, −l 0.4, −n 2). The filters used for all analyses are shown in Supplementary Table 1.

To assess the quality of SNP calling based on genomic position, we used a Python script QCvcfWindow (see Data availability) to identify the count and average quality scores of variants in 100 kb windows of the Chinook salmon data set. These metrics were plotted using Circos software (version 0.69) (Krzywinski *et al.* 2009). Homeologous regions in the Chinook salmon genome assembly were identified to better understand the influence of SNP calling in these regions. BLAST (Altschul *et al.* 1997; Camacho *et al.* 2009; Chen *et al.* 2015) was used to generate a database of the masked genome assembly and align the assembly to itself (parameters: outfmt 6, perc_identity 80, max_hsps 40000). A python script, BlastLinearFilter (parameters: gap 100000 and min 10000; see Data availability), was used to output linear alignments between homeologous regions.

### Mapping variants among species

To compare analyses among species, variants were mapped from the coho and sockeye salmon genomes to the Chinook salmon genome using a pipeline, *MapVCF2NewGenome* (see Data availability). This pipeline was also used to map sockeye salmon variants, which were on scaffolds, to a chromosome-level assembly (this assembly was submitted to the NCBI (Sayers *et al*. 2022) as version 2 of the reference genome assembly—now available as GCA_034236695.1). The sockeye salmon chromosome–mapped version was needed for different analyses (see Historical estimates of effective population size and Potentially adaptive variants).

### Coverage, relatedness metrics, and runs of homozygosity

Coverage was assessed using a Python script, *VCFStats* (see Data availability). This script finds the average depth of all SNPs per individual. It also counts the number and type of genotypes per individual. This information was used to remove individuals that had average depth/coverage less than 8× using VCFtools. A preliminary principal component analysis (PCA) had a clustering of individuals based on coverage rather than geography if they were below this threshold. These individuals were not discussed and were not analyzed further. Individuals were filtered with an average depth less than 15× depending on the analysis (noted for each analysis; Supplementary Table 1). This was because the frequency of heterozygous genotypes dropped when coverage was below 15× and some analyses were sensitive to this issue (Supplementary Table 1). Genotype information and coverage were visualized in R (R Core Team 2022) using ggplot2 (Wickham 2016).

The related package (version 0.8, parameters of coancestry: ritland = 1) (Pew *et al.* 2015) was used to calculate relatedness (Ritland 1996) in R. Values below 0 (e.g. when markers are not shared) and above 1 (e.g. when rare markers are shared) are expected with this type of estimation (Ritland 1996). There were many more private alleles in coastal populations of Chinook and coho salmon, which may have made them appear much more related. The LD-filtered SNP data sets were converted to the related format using a Python script, *VCF2Relate* (see Data availability). The reshape2 (Wickham 2007) and pheatmap (Kolde 2019) packages were used to visualize relatedness. Related individuals were included in most analyses, but we tested what would happen if related individuals were removed from PCAs. A relatedness score of 0.15 (an arbitrary value greater than what might be expected for first cousins, but less than for half-sibs—depending on the data set) was used to filter all but 1 individual from pairs that might be related. Most samples were taken as adults, and related individuals were not expected to be common within or among sampling collections.

We observed that the frequency of heterozygous genotypes depended on the SNP coverage. Genetic diversity metrics that rely on heterozygous genotypes (e.g. heterozygosity) would likely be influenced in samples with less than 15× coverage. For this reason, we included runs of homozygosity (ROH). No consistent relationship was observed between coverage and ROH. ROH were identified using PLINK (version 1.9, parameters: –homozyg –double-id, –allow-extra-chr, –homozyg-snp 25, –homozyg-kb, –homozyg-density, –homozyg-gap, –homozyg-window-het 1, –homozyg-window-snp 50, –homozyg-window-threshold 0.05, –homozyg-window-missing 5) (Chang *et al.* 2015; “PLINK 1.9”). All individuals were included in these analyses.

### Population structure and clustering analyses

PCA and admixture analyses were used to identify distinct genetic groups for each species. The admixture software requires independent variants, and PCAs can be sensitive to LD blocks, which can cause clustering that is unrelated to population structure. For these reasons, the LD-filtered SNPs were used in these analyses. To perform the admixture analyses, the LD-filtered data were converted from VCF format to a suitable format using PLINK (version 1.9, parameters: –double-id, –allow-extra-chr). We converted the chromosome names to numbers using Unix commands. The admixture analysis was performed using ADMIXTURE (version 1.3.0, parameters: –cv) (Alexander *et al.* 2009). Cluster values 1–20 were tested, and we accepted the value with the lowest cross-validation score. Admixture ancestry values were visualized in R for individuals and QGIS (QGIS.org, 2022) as an average score per sampling site.

PCAs were used to assess the groupings produced from the admixture analyses. PLINK (parameters: –pca, –double-id, –allow-extra-chr) was used to perform the PCA, and the results were visualized in R using ggplot2, reshape2, and ggrepel (Slowikowski 2021). PCAs were attempted with all the individuals, individuals with ≥15× coverage, and with highly related individuals removed. The outputs of these 3 approaches were compared to determine the influence of coverage and relatedness on clustering and the groups produced from the admixture analyses. We also verified that samples among the new (MGI) and previous data sets (Illumina) clustered together if they were from the same sampling site.

### Environmental variable PCA

To determine whether population structure was associated with environmental factors, we clustered sampling sites by environmental factors using PCA in R (parameters: prcomp, scale = T). This was visualized using the factoextra library. Each sampling site was assigned to an admixture group if the average ancestry value was ≥0.7 for a particular site. Environmental factors were taken from the WorldClim version 2.1 data set (Fick and Hijmans 2017). Elevation for each site was estimated from either Google Maps, Mapcarta, or data downloaded from the Federal Geospatial Platform (maps.canada.ca) and viewed in QGIS. Distance to the ocean was estimated as the river distance with QGIS or Google Maps.

### Historical estimates of effective population size

To estimate effective population size through time, we used the program SMC++ (parameters: -c 1000000) (Terhorst *et al.* 2017). The only mutation rate estimate for these species available at the time of writing was for coho salmon [8.0*e*−9 from (Rougemont *et al.* 2020)]. A correction was applied to this mutation rate for Chinook and sockeye salmon based on the ratio of total SNPs from these species to the total SNPs from coho salmon. For Chinook salmon, this ratio was 1.73 for the individuals subset from a similar geographic range and for the same number of individuals (mutation rate of 1.4*e*−8). For sockeye salmon, this was 0.80 for the individuals subset, with a mutation rate of 6.4*e*−9. Figures were plotted in R using ggplot2, and ocean surface temperature data were taken from previous studies (Zachos *et al.* 2008; Hansen *et al.* 2013).

### Genetic diversity

The percent of heterozygous genotypes per individual was calculated by dividing the number of heterozygous genotypes (see Coverage, relatedness metrics, and runs of homozygosity) by all genotypes (including missing data to standardize against all variants) and multiplying by 100. If missing genotypes were excluded from the calculation, values changed by 0.09% on average, and the difference ranged from 0.03 to 1.40%. Nucleotide diversity (π) and the number of polymorphic loci per location were calculated using the Stacks populations module (version 2.54, default parameters) (Catchen *et al.* 2011, 2013). The number of polymorphic loci calculated by Stacks is the number of SNPs that are variant from that sample location.

Nucleotide diversity per sampling site and *F*_st_ were both calculated from the subset of individuals with ≥15× coverage because these analyses were sensitive to coverage (i.e. as coverage increased, so did the percent of heterozygous genotypes until ≥15× coverage). *F*_st_ was calculated using VCFtools. Since nucleotide diversity was plotted per sampling site using the inverse distance weighting interpolation analysis in QGIS, other nearby sites can be used to determine if relatedness influenced nucleotide diversity regionally.

### Admixture group private alleles

A Python script, *PrivateAllele*, was used to identify private alleles among admixture groups of each species (see Data availability). Alleles were identified that were unique to each admixture group if they were present for a specified number of individuals in that group (parameter: -min 3). Individuals were assigned to an admixture group if they had ancestry values ≥ 0.7 for a particular group. Salmon with ancestry values below 0.7 were excluded from these analyses. The private allele counts were visualized in R using ggplot and the gridExtra library (Auguie 2017).

The number of individual private alleles among admixture groups was identified using another Python script, *PrivateAllelePerInd* (see Data availability). Individual private allele counts are the number of private alleles identified from a group that an individual from that group also shares. This metric identifies if there are individuals or sampling sites that were more responsible for the number of private alleles within an admixture group.

### Potentially adaptive variants

Extended haplotype homozygosity was used to identify potentially adaptive loci since we noted that ROH were not very sensitive to sequence coverage. This was important in the current study as we used samples from multiple studies that were also from several locations that might have different genotypes either due to differences in coverage or geographic distribution. The rehh library (Gautier and Vitalis 2012; Gautier *et al.* 2017) in R was used to identify extended haplotype homozygosity within and among admixture groups (assignments were made for individuals with ≥0.7 ancestry values). To perform the rehh analyses, we used SHAPEIT5 (Hofmeister *et al.* 2023) (version 5.1.1, parameter: phase_common_static) to first phase the genotypes of the MAF 0.01-filtered variants. The output was then converted to VCF format using BCFtools, and the different admixture groups were separated using VCFtools. The rehh command *data2haplohh* was then used to read in each admixture group VCF file, and the *scan_hh* command (parameters, polarized = FALSE, interpolate = FALSE) was used to calculate extended haplotype homozygosity. The within-population metric iHS was determined using the *ihh2ihs* command (default parameters), and the pairwise population metric Rsb was calculated using the *ines2rsb* command (default parameters). The *calc_candidate_regions* function (parameters: threshold = 10, window_size = 10000, min_n_extr_mrk = 8) was used to identify regions with significant differences in extended haplotype homozygosity (the Bonferroni correction *P-*value threshold was between 8.8 and 9.1 after −log10 transformation depending on species). A higher threshold was used to identify only the strongest candidates as this is only a preliminary study. We also examined overlapping 10 kb windows among all 3 species to identify candidates of potential convergent evolution.

## Results

### Coverage, ROH, and relatedness

**Fig. 2.**
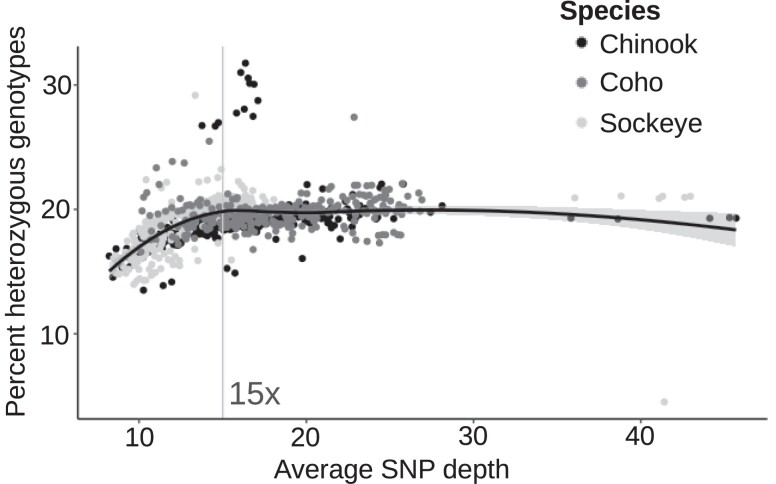
The influence of SNP coverage on heterozygous genotypes. A scatter plot of the average SNP coverage and the percent of the genotypes that were heterozygous for each salmon. The line was plotted using the loess method in ggplot. The vertical line indicates 15× coverage, used as a threshold in other analyses.

The average SNP coverage of the combined 954 salmon was 16× (Fig. 2). The average coverage of Chinook salmon was 17× (*n* = 360), coho salmon 18× (*n* = 277), and sockeye salmon 14× (*n* = 317). The percent of heterozygous genotypes for each individual appeared to depend on the SNP coverage until a depth of 15× in all species (Fig. 2). No obvious relationships among homeologous regions, SNP counts, or SNP quality scores were observed (Supplementary Fig. 3). However, quality scores and SNP counts often decreased near telomeres (Supplementary Fig. 3).

**Fig. 3.**
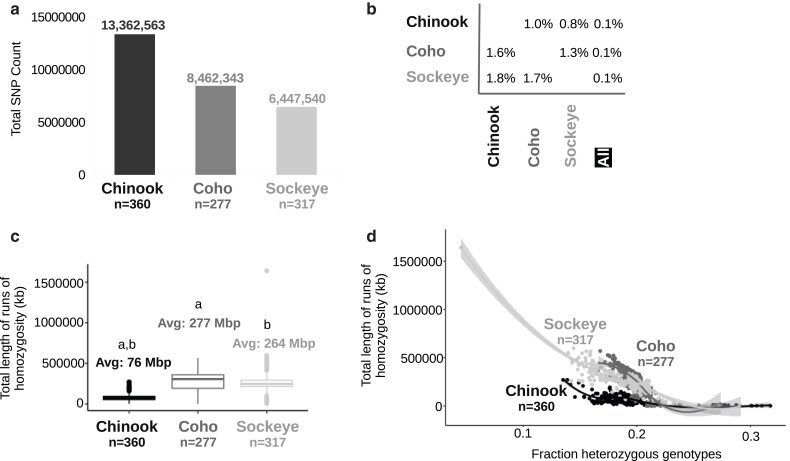
SNPs and ROH per species. a) Counts of all SNPs identified for each species with the same pipeline—differing only in score recalibration and reference genome assemblies. The number of samples per species is shown below each column. b) Percent of overlapping SNP loci among species. SNPs from sockeye and coho salmon were mapped to the Chinook salmon genome assembly. The percent of loci that overlap is shown for each species relative to the number of SNPs identified in that species (per row). The last column displays the number of loci that overlapped for all species relative to the total number of SNPs for the species in a particular row. c) Box plots of the total length of ROH for each species. Significantly different comparisons (*P* ≤ 0.05, two-tailed, Welch's *t*-test) have matching letters. The sockeye salmon with ROH > 1.5 Gb (top right) was a doubled-haploid from a previous study. d) A scatter plot of the total length of ROH and the fraction of heterozygous genotypes per individual. Lines were fitted using the loess method.

Around 6–13 million SNPs were identified per species (Fig. 3a). Chinook salmon had 1.6–2.1× more SNPs than either coho or sockeye salmon, respectively. SNP coverage did not appear to influence the difference in the number of SNPs among species. For example, the average number of heterozygous genotypes per salmon for those with 15× coverage or greater reflected the trend observed for the number of SNPs per species. These values were 2,636,167 in Chinook salmon, 1,667,558 in coho salmon, and 1,305,776 in sockeye salmon (1.6–2.0× greater in Chinook salmon). Around 0.1% of the SNPs identified in any of the species were common with both of the other 2 species (Fig. 3b). The percent of common SNPs between any 2 species ranged from 0.8 to 1.8% (Fig. 3b).

Chinook salmon had significantly shorter total lengths of ROH than the other 2 species, consistent with a higher number of SNPs (Fig. 3c). Sockeye and coho salmon had on average ∼3.5–3.6× longer total lengths of ROH than Chinook salmon. The length of ROH decreased as the fraction of heterozygous genotypes increased, but generally, sockeye and coho salmon had longer ROH when fractions of heterozygous genotypes were below 0.2 (Fig. 3d). No consistent relationship among species was observed between SNP coverage and the total length of ROH (Supplementary Fig. 4).

Coastal samples (all sites downstream of the Thompson River or outside the Fraser River—see Fig. 1) appeared highly related to each other in Chinook and coho salmon (Supplementary Fig. 5). A confounding variable was that these samples were from previous studies and sequenced with a different technology. In Chinook and coho salmon, coastal samples also had much higher counts of private alleles (see below), which is known to increase relatedness metrics (Ritland 1996). In sockeye salmon, similar high relatedness values were observed in the northern and upper Columbia River kokanee admixture groups, which also had the highest individual private allele counts (see below).

Relatedness values were generally reduced by subsetting individuals from previous studies and the current study (Supplementary Fig. 5), which would be expected if the high relatedness values were the result of increased private alleles in the coastal samples. Also, samples from the Thompson River (a tributary of the Fraser River) did not have high relatedness scores to these coastal samples in Chinook and coho salmon even though they were sequenced in previous studies. Finally, Fraser River sockeye salmon did not have a similar pattern based on sequencing technology (Supplementary Fig. 5). These 3 observations are possible if the high relatedness is associated with shared rare variants rather than a technical artifact from comparing samples from different studies.

### Population structure and clustering analyses

**Fig. 4.**
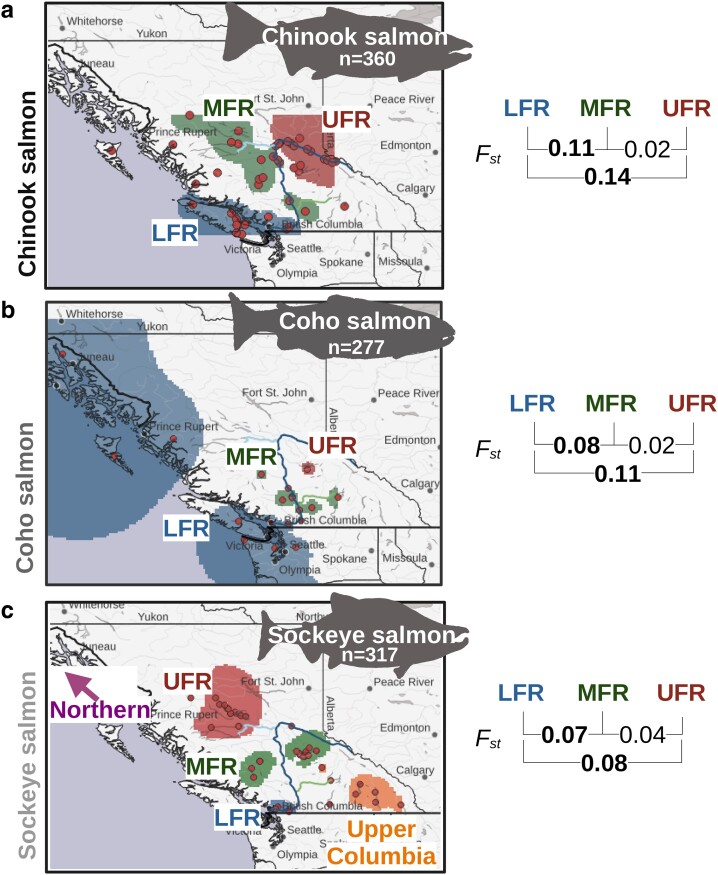
Fraser River salmon admixture groups. Map of a) Chinook, b) coho, and c) sockeye salmon admixture groups plotted in QGIS by overlaying the admixture ancestry raster plots from Supplementary Fig. 6. The Fraser River admixture groups were the LFR, MFR, and UFR. The Chinook and coho salmon LFR groups include coastal locations outside the Fraser River. Sockeye salmon have 2 admixture groups independent of the Fraser River. The northern sockeye salmon admixture group is off the map (indicated by arrow). Some sampling locations had intermediate values among admixture groups and did not have a distinct group assignment. These are the locations without background colors. Weir and Cockerham's (Weir and Cockerham 1984) *F*_st_ values are shown to the right of the maps for comparisons between admixture groups on the Fraser River. *F*_st_ comparisons between coastal (LFR) and interior clusters (MFR and UFR) are in bold text.

Each species had 3 supported Fraser River admixture groups (Table 1, Fig. 4, Supplementary Fig. 6). All groups were based on geography and were supported by PCA (Supplementary Fig. 7). These included a LFR, mid Fraser River (MFR), and UFR admixture group. We focus on the large-scale resolution of genetic groups in this study, but there was still substantial genetic variation within each of the groups identified (Supplementary Fig. 6). For sockeye salmon, there were 2 other admixture groups identified outside the Fraser River, referred to as northern and upper Columbia River kokanee (Fig. 4, Supplementary Fig. 6).

**Table 1.** Assignment of sampling locations to Fraser River admixture groups.

	LFR	MFR	UFR
Chinook salmon (*n* = 360)	**Fraser River Tributary** Chilliwack River Harrison River **Non-Fraser River** Atnarko River (MFR) Big Qualicum River Chuckwalla (MFR) Kitimat River (MFR) Little Qualicum River Marble River Nitinat River Puntledge River Quinsam River Robertson Creek Sarita River Tenderfoot Creek Yakoun River (MFR)	**Fraser River Tributary** Baker Creek (UFR) Bridge River (UFR) Elkin Creek (UFR) Endako River (UFR) Kuzkwa River (UFR) Lower Cariboo River (UFR) Nazko River (UFR) Nechako River Middle Shushwap River (LFR) Spius Creek Shovel Creek (UFR) Taseko River Upper Chilcotin River West Road River (UFR) **Non-Fraser River** Babine River (LFR)	**Fraser River Tributary** Bowron River Cottonwood River (MFR) Fontoniko Creek Holliday Creek Horsefly River (MFR) Horsey Creek Kenneth Creek McGregor River Morkill River Salmon River (MFR) Robson River Tête Jaune Torpy River Upper Cariboo River Willow River
Coho salmon^a^ (*n* = 277)	**Fraser River Tributary** Inch Creek **Non-Fraser River** Berners River Big Quilcene River Capilano River Deschutes River Kitimat River Klamath River Kwethluk River Pallant Creek Robertson Creek Tsoo-Yess River Wallace River	**Fraser River Tributary** Bridge River Chilko River Coldwater River Gates Creek Kawkawa Creek (LFR) Nahatlatch River Salmon River Seton River	**Fraser River Tributary** McKinley Creek
Sockeye salmon^b^ (*n* = 317)	**Fraser River Tributary** Cultus Lake Harrison River (MFR) Pitt River Widgeon Slough	**Fraser River Tributary** Adams Lake (LFR) Blue Lead Creek Bowron River (UFR) Chilko Lake North Chilko Lake South Chilko River Lower Horsefly River McKinley Lake Middle Horsefly River Mitchell River Quesnel Lake Taseko River Upper Horsefly River Wasko Creek	**Fraser River Tributary** Bivouac Creek Driftwood River Dust Creek Felix Creek Kuzkwa River Middle River Nadina River Paula Creek Pinchi Creek Stellako River Tachie River Takla Lake

Locations with average admixture ancestry values below 0.7 were placed in the group with the highest ancestry values and have the admixture group with the second largest ancestry value in parentheses. Bold text signifies to differentiate Fraser River Tributaries from non Fraser River bodies of water.

^a^Samples from the Big Bar Landslide were excluded since they were assigned to more than 1 admixture group.

^b^Only Fraser River admixture groups are shown for sockeye salmon (excluding northern and upper Columbia River kokanee groups).

The LFR admixture groups included sample sites in the Fraser Valley south of the Fraser Canyon (approximately where the river turns from a southern flow to a western flow before its outlet to the Pacific Ocean) (Fig. 4). In Chinook and coho salmon, the LFR admixture groups also included nearby coastal sites. For coho salmon, this included locations from Alaska to California.

We sampled fewer coho salmon north of the Fraser Valley than for the other species due to accessibility issues during their spawning season. Those north of the valley clustered in a pattern similar to Chinook salmon (i.e. MFR and UFR groups; Fig. 4). All samples, excluding those from McKinley Creek (a tributary of the Horsefly River), and some samples without known natal streams (sampled at the Big Bar Landslide), form 1 admixture group—the MFR. In Chinook salmon, the MFR admixture group is formed from the collection of sites west of the Horsefly River and the Nechako River south. This geographic region is known as the Interior Plateau. In sockeye salmon, bodies of water from the Chilcotin and Quesnel River watersheds make up the MFR admixture group (Fig. 4).

The UFR coho and Chinook salmon admixture groups include most of the Quesnel River watershed locations, except the lower Cariboo River near the confluence with the Fraser River (Fig. 4). For coho salmon, this was from a single known sampling location and potentially other locations from salmon that were collected at the Big Bar Landslide. The Chinook salmon UFR group also included sampling sites above the Nechako River (in or near Robson Valley, a part of the Rocky Mountain Trench). The UFR sockeye salmon admixture group is comprised of Nechako River watershed sites.

The LFR admixture groups, in all species, have the greatest genetic differentiation (i.e. *F*_st_) from the other admixture groups (Fig. 4). In addition, the UFR admixture groups have higher differentiation from the LFR admixture groups than the MFR groups (Fig. 4). *F*_st_ values were generally much lower between the UFR and MFR admixture groups.

### Environmental factors influencing genetic structure

**Fig. 5.**
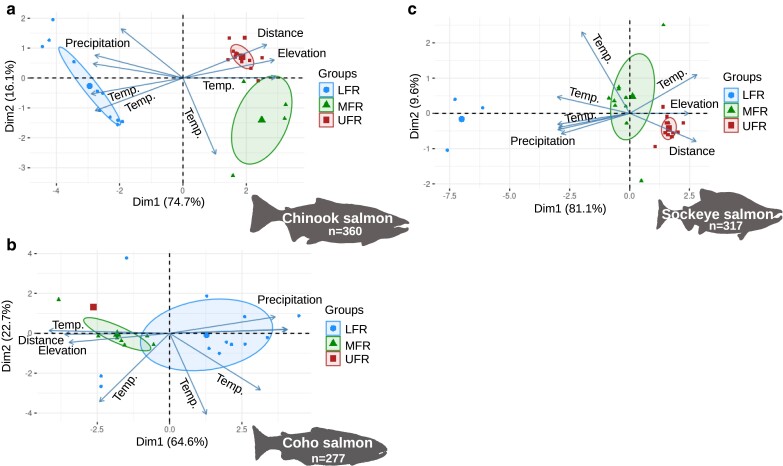
Clustering of admixture groups by environmental factors. Biplots of environmental factors (arrows) used in PCAs of sample locations (points) of a) Chinook, b) coho, and c) sockeye salmon. The environmental factors included those from WorldClim version 2.1, elevation, and distance to the ocean. Environmental factors were simplified as temp. (including annual mean temperature, minimum temperature of the coldest month, maximum temperature of the warmest month, and temperature range), precipitation (including driest month precipitation, wettest month precipitation, and annual precipitation), elevation, and distance to the ocean. These variables were not simplified in Supplementary Fig. 8. Locations were colored by their assignment to admixture genetic groups, but no genetic data were used to produce these PCAs. Only sample sites that had average ancestry values ≥ 0.7 from the LFR, MFR, and UFR genetic groups are shown for simplicity (see Supplementary Fig. 8 for all locations). Each small symbol represents a sampling site, and the larger symbol represents the center of the ellipse if there were enough points to calculate and draw the ellipse (ellipse level 0.4). The LFR group includes coastal sites outside the Fraser River for Chinook and coho salmon.

Sample locations from the same admixture group were clustered by environmental factors in a PCA (Fig. 5, Supplementary Fig. 8). This clustering was performed only with environmental factors, and it emphasizes the different environments the admixture groups are exposed to. Sites with predominately LFR ancestry values, for example, were in regions with higher annual precipitation and higher mean annual temperatures (Supplementary Fig. 8). Environmental factors related to temperature range, the maximum temperature during the warmest month, elevation, and distance to the ocean were major factors influencing the separation of the MFR and UFR clusters (Supplementary Fig. 8). While there was some overlap between MFR and UFR admixture groups, there were still distinct environmental factors between them overall. This was the case for all 3 species.

### Historical estimates of effective population size

**Fig. 6.**
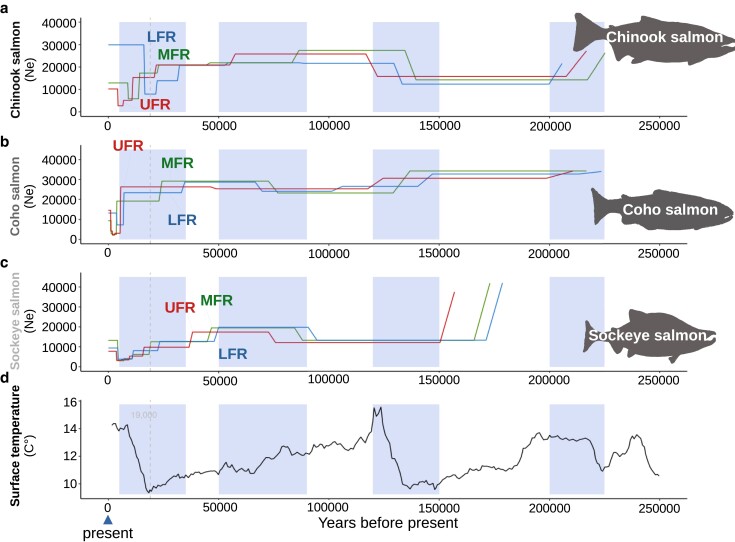
Estimated historical effective population size of Fraser River salmon. The effective population size of a) Chinook, b) coho, and c) sockeye salmon estimated using SMC++. Only 1 sampling site from each Fraser River admixture group was retained for clarity. Supplementary Fig. 9 contains all sites. The estimated last glacial maximum of around 19,000 years ago (Clark *et al.* 2009) is shown with a dashed vertical line. Other periods of time were highlighted when there were major changes in effective population size for more than 1 species. d) Estimates of ocean surface temperatures for the same timeline (Zachos *et al.* 2008; Hansen *et al.* 2013).

Historical estimates of effective population size (*N*_e_) are strikingly different among species (Fig. 6, Supplementary Fig. 9). Prior to 200,000 years before the present, almost all Chinook salmon sampled experienced drops in estimates of effective population size (Fig. 6, Supplementary Fig. 9). This coincides with a time when there was an increase in ocean surface temperatures (Fig. 6d).

**Fig. 7.**
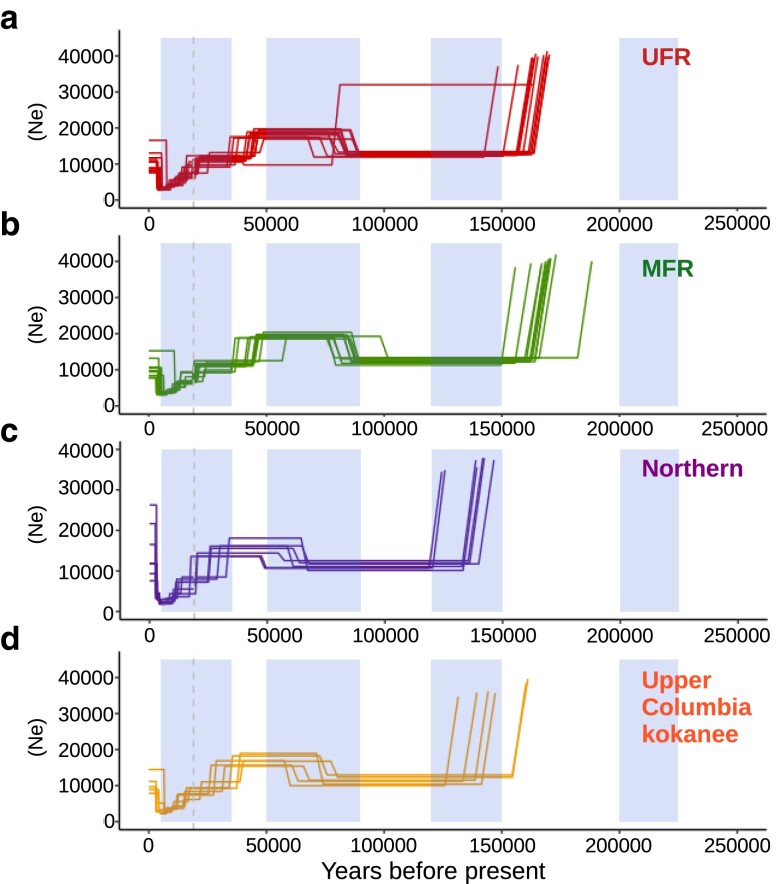
Estimated historical effective population sizes of some admixture groups of sockeye salmon. The effective population size of sockeye salmon sampling locations from the a) UFR, b) MFR, c) northern, and d) upper Columbia River kokanee admixture groups.

Between 120,000 and 150,000 years before the present, coho and some sockeye salmon experienced major declines in estimated effective population size (Figs. 6 and 7). In other sockeye salmon, the decline was earlier—between 150,000 and 200,000 years ago. There are distinct times when these decreases occurred in sockeye salmon based on location (Fig. 7). For some sockeye salmon locations and most coho salmon sites, the drop in effective population size occurred around the penultimate glacial maximum, when ocean temperatures were at their lowest. Neither species recovered to previous estimates of effective population size for over 100,000 years (Fig. 6 and Supplementary Fig. 9). This is consistent with the decrease in polymorphic loci and increases in ROH compared to Chinook salmon. Interestingly, the Chinook salmon effective population size increased during this same time frame. A similar reversal among species was observed between 50,000 and 90,000 years before the present, where there were increases in estimates of effective population size for coho and sockeye salmon, but decreases in Chinook salmon (Fig. 6, Supplementary Fig. 9).

In all species, there were decreases in effective population size around the last glacial maximum, 19,000 years before the present (Figs. 6 and 7, Supplementary Fig. 9). The timing of these decreases in effective population size varied in time by species, admixture group, and sampling sites within admixture groups (Figs. 6 and 7, Supplementary Fig. 9). Most demographic histories have a step pattern near or after the last glacial maximum. Some coastal Fraser River groups have earlier decreases in effective population sizes than the other Fraser River groups. The exception is LFR sockeye salmon sites, which have similar or more recent declines in population size than MFR and UFR locations (Supplementary Fig. 9).

### Understanding the genetic diversity of Fraser River salmon

**Fig. 8.**
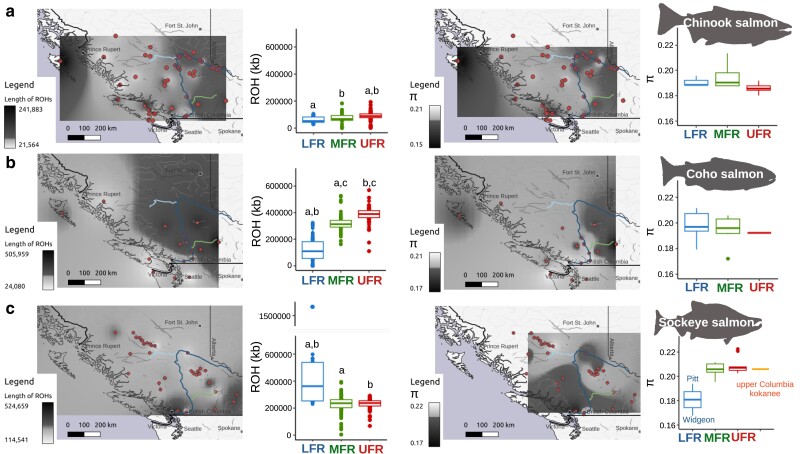
Genetic diversity of Fraser River salmon. Genetic diversity metrics of a) Chinook, b) coho, and c) sockeye salmon. (Left) Map of total length (kb) of ROH averaged for each sampling site. This analysis included all individuals and was mapped in QGIS using the inverse distance–weighted interpolation method. The scale is quantile-based. (Middle-left) Box plots of the total length of ROH for samples assigned to the different admixture groups with ancestry values ≥ 0.7. Significantly different comparisons (*P* ≤ 0.05, two-tailed, Welch's t-test) have matching letters. (Middle-right) Map of nucleotide diversity (π) of each sampling site, excluding salmon with less than 15× SNP coverage. The scale is quantile-based. (Right) Nucleotide diversity box plots of sampling sites with average admixture ancestry values ≥ 0.7 from individuals with at least 15× SNP coverage. There were no significant differences in comparisons of nucleotide diversity. For Chinook and coho salmon, the LFR groups included samples outside the Fraser River.

On average, total ROH were significantly shorter in LFR locations than MFR and UFR sites of Chinook and coho salmon (Fig. 8). Lower ROH is a proxy for greater genetic diversity, suggesting that the Chinook and coho salmon LFR admixture groups have higher diversity than the MFR and UFR groups. Sample sites with the longest average total ROH were the Yakoun River (Haida Gwaii) and Salmon River (a tributary of the Thompson River) for Chinook and coho salmon, respectively (Supplementary Fig. 10). Locations with the shortest average total ROH (i.e. highest diversity) for Chinook and coho salmon, respectively, were the upper Chilcotin River (a tributary of the Fraser River) and the Deschutes River (a tributary of the Columbia River) (Supplementary Fig. 10). ROH were the most variable in the Morkill and upper Chilcotin River locations for Chinook salmon. The individuals with the shorter ROH in the upper Chilcotin River drove the average to the lowest for Chinook salmon.

There was a reverse trend for ROH in sockeye salmon compared to Chinook and coho salmon (Fig. 8). Most of the sockeye salmon sites with the longest ROH (i.e. the lowest diversity) were from the LFR (i.e. Cultus Lake, Pitt River, and Widgeon Slough). Cultus Lake and Widgeon Slough have small population sizes, often under 1,000 salmon in recent generations (DFO 2020; Doutaz *et al.* 2023).

The Harrison River is a LFR tributary, and although not a part of the LFR admixture group due to being below the 0.7 admixture threshold, it had the lowest average ROH of all sockeye salmon collections (Supplementary Fig. 10). The Harrison River, in the early 1980s, had around 300,000 spawning adults (Doutaz *et al.* 2023). This suggests that the low genetic diversity in the LFR admixture group might be a result of sampling or due to recent declines in population size.

A confounding variable is that Widgeon Slough and possibly the Harrison River were the only samples of the sea-type ecotype taken from the Fraser River (Beacham and Withler 2017; Doutaz *et al.* 2023). All of the other Fraser River samples were likely lake-type. Sea and lake-type sockeye salmon migrate to the ocean, unlike kokanee, but they have distinct life histories from each other (Wood *et al.* 2008). Also, some Harrison River salmon are sea-type and some are lake-type.

Nucleotide diversity (π), a population level metric rather than an individual level metric like ROH, had a more complex pattern. Nucleotide diversity ranged from 0.15 (Chinook salmon—Yakoun River) to 0.22 (sockeye salmon—Pinchi Creek). None of the π comparisons among admixture groups were significantly different from each other for any of the species. We discuss π for comparison to ROH and as a reference for other studies.

Most Chinook salmon locations had π values between 0.18 and 0.20. However, the Yakoun River (Haida Gwaii) had the lowest π of all sites for Chinook salmon (π = 0.15). Many sampling locations with the highest nucleotide diversity were from the MFR (Fig. 8). The Fraser River Chinook salmon sampling sites had average π values similar to coastal locations along the British Columbia coast (Fig. 8).

Coho salmon ranged in π from 0.17 to 0.21, with the lowest collection site being the Salmon River in the South Thompson River drainage and the highest being the Kawkawa Creek below the Fraser Canyon (Fig. 8). Fraser River coho salmon had comparable or higher levels of π than those from California to Alaska. The most extreme northern (Kwethluk River) and southern sites (Klamath River) had the second and third lowest π, respectively.

All sockeye salmon sampling locations had π above 0.19 except for Widgeon Slough, which was 0.17. Cultus Lake might have had lower π than Widgeon Slough based on its longer ROH, but this location was excluded from this analysis since it had lower SNP coverage than 15×. Similarly, most northern coastal and upper Columbia River samples were excluded from the nucleotide diversity analysis because they had SNP coverage below the threshold.

The number of polymorphic loci per location, a metric that measured the number of loci that were polymorphic in each sample location separately, was highest in Chinook salmon sampling sites (Supplementary Fig. 11). This was followed by coho and sockeye salmon (Supplementary Fig. 11). This order is consistent with the total number of SNPs identified in each species (Fig. 3). While the number of polymorphic loci depended on the number of samples per location, it remained higher in Chinook salmon at comparable sample counts per location (Supplementary Fig. 11). Even though Chinook salmon had the greatest number of polymorphic loci per location, π was often lower in Chinook salmon than for coho and sockeye salmon locations (Supplementary Fig. 11). This is consistent with higher individual percent heterozygous genotypes in some coho and sockeye salmon at comparable SNP coverages (Fig. 2).

### Admixture group private alleles

**Fig. 9.**
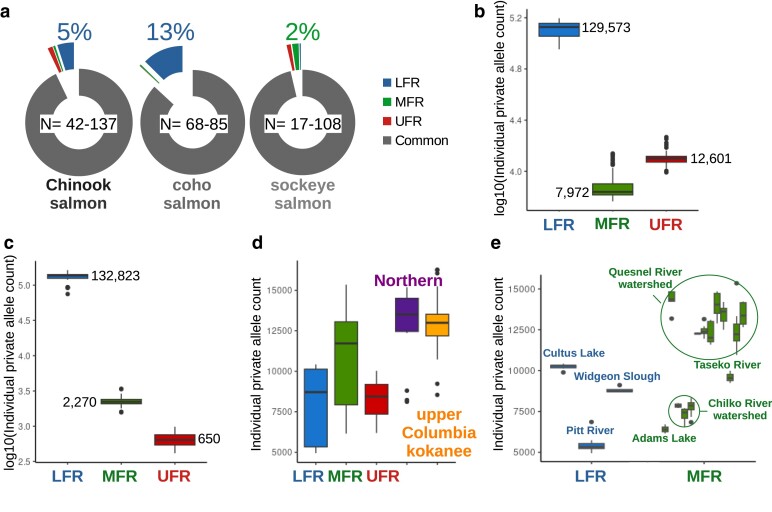
Private allele analyses of admixture groups. a) Pie charts of the percent of SNPs with private alleles from 1 of the 3 Fraser River admixture groups (only individuals with ≥0.7 admixture ancestry values were used). For Chinook and coho salmon, the LFR groups included bodies of water outside the Fraser River watersheds. The range of samples per admixture group is shown in the middle of each pie chart. Box plots of individual private allele counts (i.e. the number of private alleles identified from a group that an individual from that group also shares) of b) Chinook, c) coho, and d) sockeye salmon from each admixture group. Average values were plotted for Chinook and coho salmon admixture groups since the *y*-axis was plotted with a log10 scale due to the large difference among admixture groups of these species. The only comparisons that were not significantly different at *P ≤* 0.05, using a two-tailed Welch's t-test, were the sockeye salmon LFR vs. UFR and northern vs. upper Columbia kokanee admixture groups. e) Box plots of sockeye salmon individual private allele counts of bodies of water from the LFR and MFR admixture groups.

There were more private alleles identified in the LFR admixture groups of Chinook and coho salmon than the other admixture groups, with 5–13% of all the identified SNPs being private to the LFR groups (Fig. 9a). Sockeye salmon had a more even distribution of private alleles among admixture groups than Chinook or coho salmon (Fig. 9a). However, the sample size of the LFR sockeye salmon group was the lowest of all comparisons (*n* = 17), and this may have impacted the number of private alleles identified. Also, sockeye salmon from the Harrison River (in the LFR region) had an average admixture ancestry value below 0.7 and were excluded from this analysis. This site had greater genetic diversity metrics than most other sockeye salmon locations (see previous section), and the exclusion of this river would be expected to reduce the observed private alleles from this region.

Analyses of individual private alleles (the number of private alleles shared by the admixture group and an individual from that group) of Chinook and coho salmon provided more evidence that LFR individuals had more unique alleles than individuals from MFR and UFR groups (Fig. 9b and c). Each LFR individual had roughly 130,000 private alleles not found in the other admixture groups. Other admixture groups had much fewer individual private alleles compared to LFR salmon.

Sockeye salmon did not have a comparable admixture group to the LFR group in Chinook and coho salmon with a large store of private alleles. The northern and upper Columbia River kokanee admixture groups had the highest average individual private allele counts (Fig. 9d). Most sampling locations in the Fraser River have lower individual private alleles compared to those outside the Fraser River. The main exceptions are samples from the Quesnel River watershed, which have similar values as those outside of the Fraser River (Fig. 9e).

### Potentially adaptive variants

Twenty candidate adaptive loci were identified in pairwise comparisons among admixture groups based on expanded haplotype homozygosity analyses (Supplementary Fig. 12, Table 2, Supplementary File 3). No significant loci were identified within admixture group analyses (iHS metric). Most candidate loci were identified among Chinook salmon admixture groups (14 out of 20). Twelve of these loci overlapped with the boundary of a gene (Table 2). Two homologs of the *trace amine-associated receptor 13C* (TAAR13C) gene overlapped with candidate loci identified in comparisons of admixture groups of sockeye salmon (Supplementary Fig. 12, Table 2). One of these genes was identified by comparing the LFR and MFR groups. The other was identified from the comparison of the LFR group and the UFR group.

**Table 2.** Significant extended haplotype homozygosity among admixture groups.

Species	Admixture comparison LFR vs. MFR	Admixture comparison LFR vs. UFR	Admixture comparison MFR vs. UFR	Nearest gene
Chinook salmon	LG09:14.36–14.37	LG09:14.36–14.37		CSGALNACT1^a^
	LG10:5.22–5.23			ENDOD1^a^
	LG11:36.37–36.38			MACO2^a^
	LG21:43.68–43.69			CTNNA1
			LG01:54.33–54.34	HCA2
			LG07:46.47–46.48	SRGAP3^a^
			LG10:2.25–2.26	AIMP1B^a^
			LG13:37.80–37.81	EPHA7^a^
			LG13:37.98–37.99	LOC121838999
			LG18:25.30–25.31	METTL24^a^
			LG18:33.39–33.40	FOSL2
			LG25:28.04–28.05	LRPPRC^a^
			LG26:1.26–1.27	KLF12B^a^
			LG30:17.88–17.90	MINK1
Coho salmon	LG18:24.62–24.63			ASB2^a^
			LG10:24.15–24.16	PTPRD
Sockeye salmon	LG26:45.73–45.74			TAAR13C^a^
		LG09:23.72–23.73		TAAR13C^a^
			LG11:31.22–31.53	Two genes overlap
			LG04:28.48–31.57	Many genes

All locations are relative to the Chinook salmon genome assembly for comparison (see Supplementary File 3 for species-specific information). Format of location: Chinook salmon linkage group: start (distance in Mb) to end (distance in Mb).

^a^Location overlaps with gene.

To identify if there were potential adaptive variants shared among species, we searched for overlapping regions with high −log10 *P-*values (we were searching for signals of convergent evolution with this analysis). The overlap with the highest −log10 *P-*value was 3.6. The chance of having an overlap with this value was expected by random at least twice by chance given the distribution of *P-*values. These overlaps were not considered significant and are not shown or discussed below.

## Discussion

While there have been many studies to examine population structure in the Fraser River (Small *et al.* 1998; Teel *et al.* 2000; Withler *et al.* 2000; Beacham *et al.* 2001, 2003, 2006b, 2006a, 2017; Nelson *et al.* 2001; Beacham and Withler 2017; Xuereb *et al.* 2022), this is the first to use whole-genome resequencing data for a large distribution of multiple salmon species. The improved resolution of resequenced genomes allowed us to compare the genetic diversity among salmon species, calculate nucleotide diversity at the genome level, and evaluate historical influences on population structure. These types of analyses would suffer using fewer genetic markers. As an example, genetic and nucleotide diversity estimates are dependent on the nucleotide variants used to estimate them (e.g. polymorphic loci in 1 region might not be polymorphic in another), and whole-genome analyses allow unbiased estimates among locations. These genome sequences also help us to capture a better snapshot in time for comparisons in future studies.

### Species-level differences of polymorphic loci

Genetic diversity can be understood at both a species and population level. We first discuss the diversity at a species level and will return to the population level later. We expected different levels of polymorphic loci among Pacific salmon based on previously published research (Allendorf and Utter 1979). Several studies observed that sockeye salmon, and sometimes coho salmon, have the lowest levels of polymorphic loci or average heterozygosity among the Pacific salmon (Utter *et al.* 1973; Allendorf and Utter 1979; Wood *et al.* 1994).

Our findings indicate that Chinook salmon have ∼1.6–2.1× the number of polymorphic loci and a corresponding drop in the length of ROH when compared to coho or sockeye salmon (∼27–29% of the ROH length). We observed the same trend based on the total number of heterozygous genotypes among the species when only looking at individuals with higher than 15× coverage. While sequencing coverage likely impacts the number of polymorphic loci identified, the scale of the differences we observed, the greater geographic sampling distance covered by the species with fewer polymorphic loci, a similar trend in ROH, and a similar trend with heterozygous genotypes of individuals with at least 15× coverage supports that these findings are robust. If we assume that salmon species had similar levels of genetic diversity during speciation and that mutation rates are comparable, coho and sockeye salmon likely had a greater reduction in standing genetic variation than Chinook salmon.

From modeling of effective population size through time, there is evidence that sockeye and coho salmon experienced a large drop in effective population size around the penultimate glacial maximum that Chinook salmon did not experience (Chinook salmon did experience an earlier drop in effective population size, but the size rebounded after ∼50,000 years). We must consider that we do not have precise estimates of mutation rates for all of these species. Estimated mutation rates ranged from 6.4*e*−9 to 1.4*e*−8 in the current study. An estimate of the mutation rate in Atlantic salmon (*Salmo salar*), a closely related species, was 4.3*e*−9 (Bergeron *et al.* 2023), which is just lower than the estimate for sockeye salmon in the current study. Even without precise dates or effective population size estimates, however, the drop in effective population size did not rebound to prior levels in sockeye and coho salmon as it did in most Chinook salmon sites. This could have resulted in a loss of polymorphic loci in both sockeye and coho salmon.

While the SNP count, effective population size, and ROH data are consistent with this hypothesis, the nucleotide diversity analysis appears to be inconsistent. For example, why would sockeye and coho salmon have similar nucleotide diversity if Chinook salmon have more polymorphic loci overall (sockeye salmon average π: 0.21, coho salmon: 0.20, and Chinook salmon: 0.19)? Simulation studies may be useful in uncovering the possible origins of these results.

### Influences on the population structure of Fraser River salmon

Turning from the species level to the population level, researchers have known the basic population structure of Fraser River salmon since the mid-1990s to the mid-2000s (Wood *et al.* 1994; Small *et al.* 1998; Teel *et al.* 2000; Withler *et al.* 2000; Nelson *et al.* 2001; Beacham *et al.* 2003, 2006a, 2006b, 2017; Beacham and Withler 2017; Xuereb *et al.* 2022). This knowledge was based on relatively few genetic markers but often many samples from a wide distribution. In the current work, we sampled a moderate number of locations and individuals but resequenced entire genomes. The basic population structure identified in the Fraser River from resequencing genomes was similar to the findings from earlier studies using microsatellite and allozyme genetic markers (e.g. lower, mid, and upper groups), except the Thompson River groups were missing since we did not sample the Thompson River drainage enough to reconstruct them. We interpret the consistency among studies to mean that these genetic clusters are stable through time [as sampling took place at various times, even within studies (Beacham *et al.* 2003, 2006a)], the clusters are robust since they can be identified with only a few genetic markers, and the entire genome is impacted by these groupings.

Understanding why we observe these genetic groups is important [e.g. Nadeau *et al.* (2016) and Rougemont *et al.* (2023)]. Are the admixture groups technical artifacts from trying to cluster genomes influenced by isolation-by-distance from a single population? Are they artifacts from the salmon transfers between regions of the Fraser River (Withler 1982)? Were they formed as a result of different colonization events (or a combination of events)? Are they a consequence of environmental hurdles to gene flow? Were they formed from adaptations to specific environmental conditions?

If the groups were technical artifacts from trying to cluster individuals from a gradient caused by isolation-by-distance of a single population, they might not be useful representations of biologically meaningful groups from a conservation or management perspective. If instead, groups were shaped by different colonization events, they might reflect those colonization histories rather than the environments they occupy. If the groups resulted from limited gene flow, we might expect the salmon to have specific adaptations to environmental factors that influence gene flow (e.g. the ability to pass regions of the Fraser River such as the Fraser Canyon), but not necessarily to other environmental conditions. These are only some examples of why these groups might exist. The genetic groups could also be influenced by a combination of these processes.

Previous researchers have suggested that isolation-by-distance is an important factor influencing salmon genetics (Withler *et al.* 2000; Rougemont *et al.* 2020, 2023). Others have suggested that different colonization events (e.g. from different glacial refugia) influence population structure in Fraser River salmon (Wood *et al.* 1994; Small *et al.* 1998; Teel *et al.* 2000; Withler *et al.* 2000). Still others have suggested that barriers to gene flow like the Fraser Canyon (specifically Hell's Canyon) are important for population structure (Wehrhahn and Powell 1987). Finally, researchers have also suggested that genetic adaptations to environmental conditions were important (Small *et al.* 1998). Analyzing multiple species can help distinguish which hypotheses are more supported (Nadeau *et al.* 2016).

While we observed lower *F*_st_ between neighboring admixture groups in all species, which would be consistent with isolation-by-distance, we also observed that private alleles were more common in the UFR group of Chinook salmon than in the MFR group. If populations were delineated strictly by isolation-by-distance, we might expect UFR salmon to have the lowest count of private alleles. This is because the UFR region would only have gene flow from the MFR in this model and could only have a subset of alleles from the MFR. Other models might be used to account for this inconsistency (e.g. different colonization events or recent population declines in the MFR population).

Also, in all 3 species, sampling sites of an admixture group were clustered together based on environmental factors in a PCA. If admixture groups were the result of neutral genetic variation or different colonization events alone, we would expect random sorting of groups or for it to be based on the distance between locations. This suggests that the environment is a major factor influencing genetic variation in Fraser River salmon, but still does not exclude isolation-by-distance or separate colonization events.

Indeed, from demographic modeling, separate colonization events appear to have been an important source of sockeye salmon in the Fraser River and possibly for Chinook salmon. For sockeye salmon, there are at least 2 unique demographic histories, one common to LFR sites and the other to MFR and UFR locations. These groups have unique demographic histories starting as early as the penultimate glacial maximum. In Chinook salmon, LFR sampling locations have an earlier decrease in effective population size that may be indicative of an earlier colonization history. Overall, the variability in the demographic histories of the species suggests a complex colonization history.

In all 3 species, the LFR admixture group was substantially different from the other admixture groups. The LFR had the highest *F*_st_ values among admixture groups, and 5–13% of the total Chinook and coho salmon SNPs were private alleles of the LFR admixture group. If we also consider the velocity barriers between these groups (Wright 2022), we have several pieces of evidence to suggest a substantial gene flow and migration barrier due to the Fraser Canyon. In support of this hypothesis, longer ROH in coho salmon appeared to be demarcated near Hell's Canyon in the Fraser Canyon. If the Fraser Canyon is a migration barrier, we would expect to observe these genetic delineations in all species, and generally, this is what was observed.

In a study of Fraser River sockeye salmon, researchers observed possible adaptations to environmental differences among populations (Eliason *et al.* 2011). They observed that coastal populations had significantly different cardiac morphology and performance from other groups and that cardiac morphology was correlated with migration difficulty. River temperature was also correlated with cardiac performance. This study highlights the importance of the environment on population structure and migration difficulty and relates the genetic structure among admixture groups to phenotypic variation. We note that in the comparison of extended haplotype homozygosity between the LFR coho salmon with the MFR group, a potential adaptive locus was identified that overlapped with the *Ankyrin Repeat And SOCS Box Containing 2* (ASB2) gene. This gene is thought to be involved in heart development (Yamak *et al.* 2020; Min *et al.* 2021).

### Influence of adaptation on the population structure of Fraser River salmon

When comparing genetic groups, we identified 20 candidate adaptive loci. No significant loci overlapped among species, which would have been evidence for convergent evolution. Rather than discuss all 20 regions, we will focus on what these loci reveal in general and, as an example, discuss the olfactory receptor gene TAAR13C. Potentially adaptive loci overlapped with 2 TAAR13C genes and were identified in separate comparisons of the LFR sockeye salmon with MFR and UFR groups.

In general, adaptive loci among the genetic groups reveal different selective pressures along the Fraser River drainage. While the environmental PCA gave us insight into how genetic groups were organized based on environmental components, adaptive loci can reveal how these and other elements shape the genomes of salmon through generations. In the type of analysis we used, we did not need to know what these elements were. This means we can discover adaptation caused by unknown and unexamined factors.

We may be able to formulate a hypothesis regarding a mode of adaptation in the case of the TAAR13C genes. The olfactory receptor, TAAR13C, is directly involved in detecting putrescine, cadaverine, and other diamines (typically associated with decomposing flesh) (Hussain *et al.* 2013; Tessarolo *et al.* 2014; Liberles 2015; Gainetdinov *et al.* 2018). The TAAR13C gene has also previously been found to be associated with sea age at maturity in Atlantic salmon (Sinclair-Waters *et al.* 2022) and to possibly be under selection in another study of Atlantic salmon (not peer-reviewed at the time of writing) (Miettinen *et al.* 2023). Since the TAAR13C gene appears to be involved in the timing of maturation, 1 hypothesis is that diamines could act as a signal for maturation for the different genetic groups of sockeye salmon in the Fraser River. One source of diamines to consider for this hypothesis comes from eggs. A study of Arctic charr (*Salvelinus alpinus*) eggs and alevin revealed that during these developmental stages, different amounts of putrescine and cadaverine were produced during the spawning season (Srivastava *et al.* 1992). If diamines influenced the maturation of MFR and UFR groups of sockeye salmon, this information would be valuable in conservation and management. Understanding the modes of adaptation of other potential adaptive loci could likewise be useful for these purposes.

## Conclusion

From analyzing hundreds of resequenced genomes of Chinook, sockeye, and coho salmon, mostly from the Fraser River, we identified genetic groups that had previously been identified with only a few microsatellites. With data from resequenced genomes, we were able to examine how these groups might have formed. They appear to have been influenced by many factors, including isolation-by-distance, migration barriers, separate glacial refugia in sockeye salmon, and the diverse environmental factors of the Fraser River drainage. We identified 20 potentially adaptive loci among the different groups of salmon, which is indicative of unique patterns of selection in the various regions of the river. Two of these loci overlapped with homologs of the TAAR13C gene. This gene could be an important target for future studies, to investigate if there is a link between it and timing of maturation in Fraser River sockeye salmon. Finally, by examining 3 species, we were able to identify commonalities and differences. Patterns of historical effective population size were dramatically different among the 3 species and could explain the variable genetic diversity currently observed among them. In terms of information useful from a conservation and management perspective, we have generated resequenced genomes that can be reused in future studies, determined metrics for comparison, and identified loci that could impact the timing of maturation of salmon in the Fraser River.

## Supplementary Material

jkae169_Supplementary_Data

## Data Availability

Resequenced genomes from this study are available on the NCBI (coho salmon: PRJNA986075, sockeye salmon: PRJNA930425, and Chinook salmon: PRJNA694998 and PRJNA1090956). Truth SNP data sets used for SNP calling are available in Supplementary File 2. SNP data sets are available in figshare (https://doi.org/10.25387/g3.25705428). Scripts are available at github.com/KrisChristensen (repositories: PrivateAllelePerInd, PrivateAllele, VCF2Relate, VCFstats, QCvcfWindow, BlastLinearFilter, and MapVCF2NewGenome). Supplemental material available at G3 online.
