# Supplementary material for: Revealing the evolutionary history and contemporary population structure of Pacific salmon in the Fraser River through genome resequencing
Source: G3 (Bethesda). 2024 Jul 23;14(10):jkae169. doi: 10.1093/g3journal/jkae169 (PMC11457079; doi:10.1093/g3journal/jkae169)
Supplement: jkae169_Supplementary_Data [file jkae169_supplementary_data.zip › Figure_S2_G3-2024-405247.pdf]

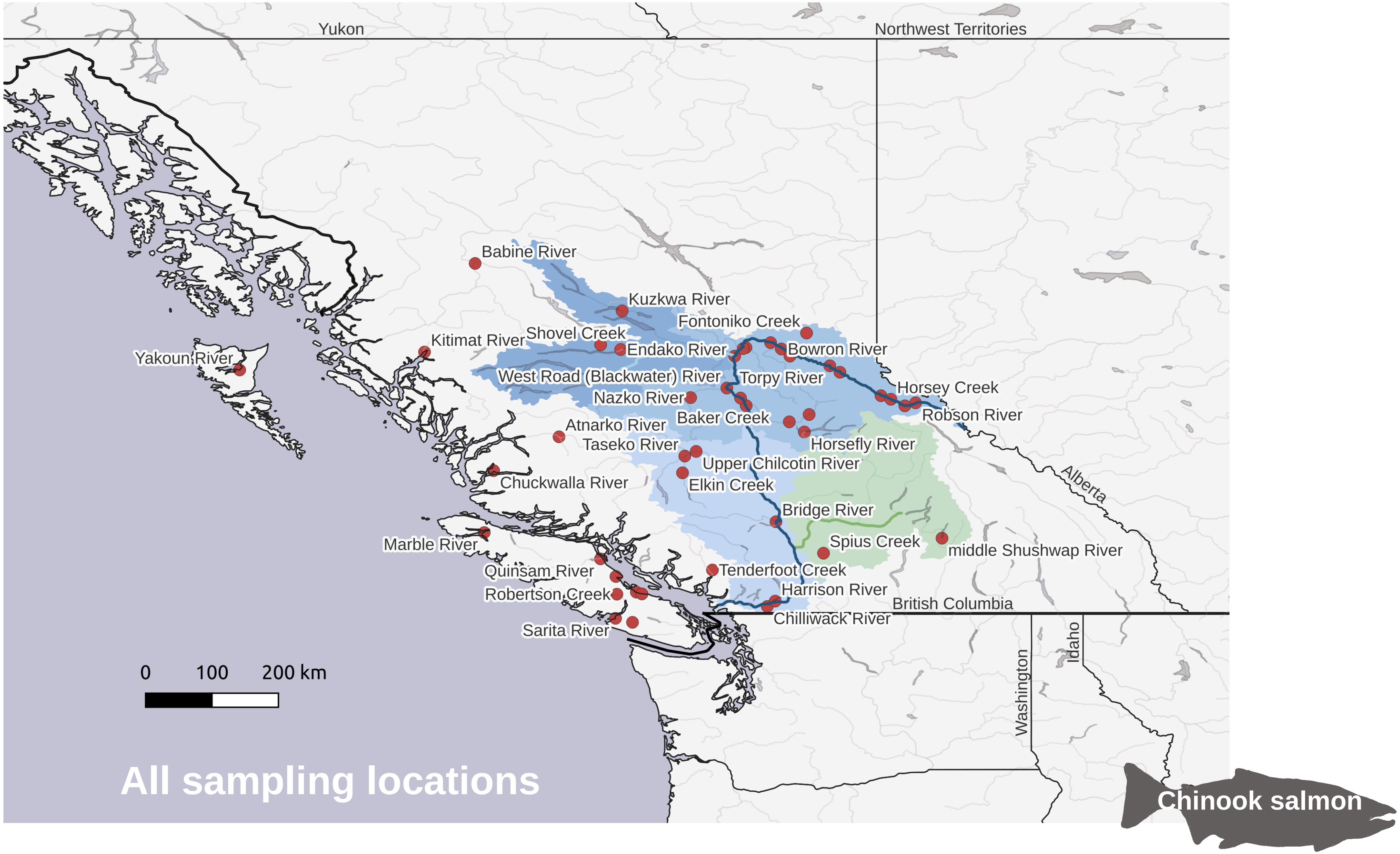
Yukon
Northwest Territories
Babine River
Kuzkwa River
Fontoniko Creek
Kitimat River
Shovel Creek
Endako River
Bowron River
Yakoun River
West Road (Blackwater) River
Torpy River
Horsey Creek
Nazko River
Robson River
Atnarko River
Baker Creek
Taseko River
Horsefly River
Upper Chilcotin River
Chuckwalla River
Elkin Creek
Alberta
Bridge River
Marble River
Spius Creek
middle Shushwap River
Quinsam River
Tenderfoot Creek
Harrison River
Robertson Creek
British Columbia
Chilliwack River
Sarita River
Washington
Idaho
0
100
200 km
All sampling locations
Chinook salmon

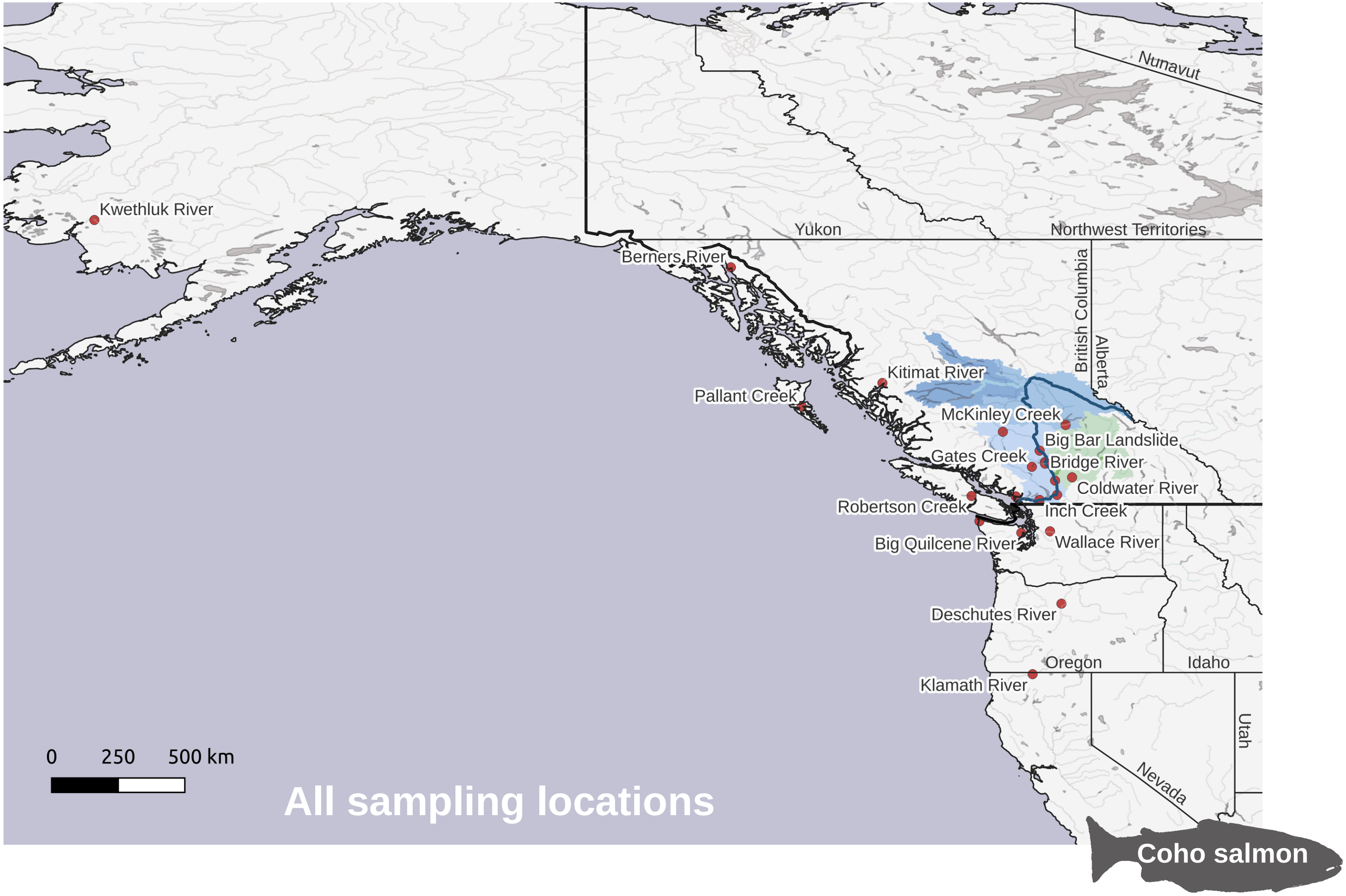
Kwethluk River
Yukon
Northwest Territories
Nunavut
Berners River
British Columbia
Alberta
Kitimat River
Pallant Creek
McKinley Creek
Big Bar Landslide
Gates Creek
Bridge River
Coldwater River
Robertson Creek
Inch Creek
Big Quilcene River
Wallace River
Deschutes River
Oregon
Idaho
Klamath River
Utah
Nevada
0 250 500 km
All sampling locations
Coho salmon

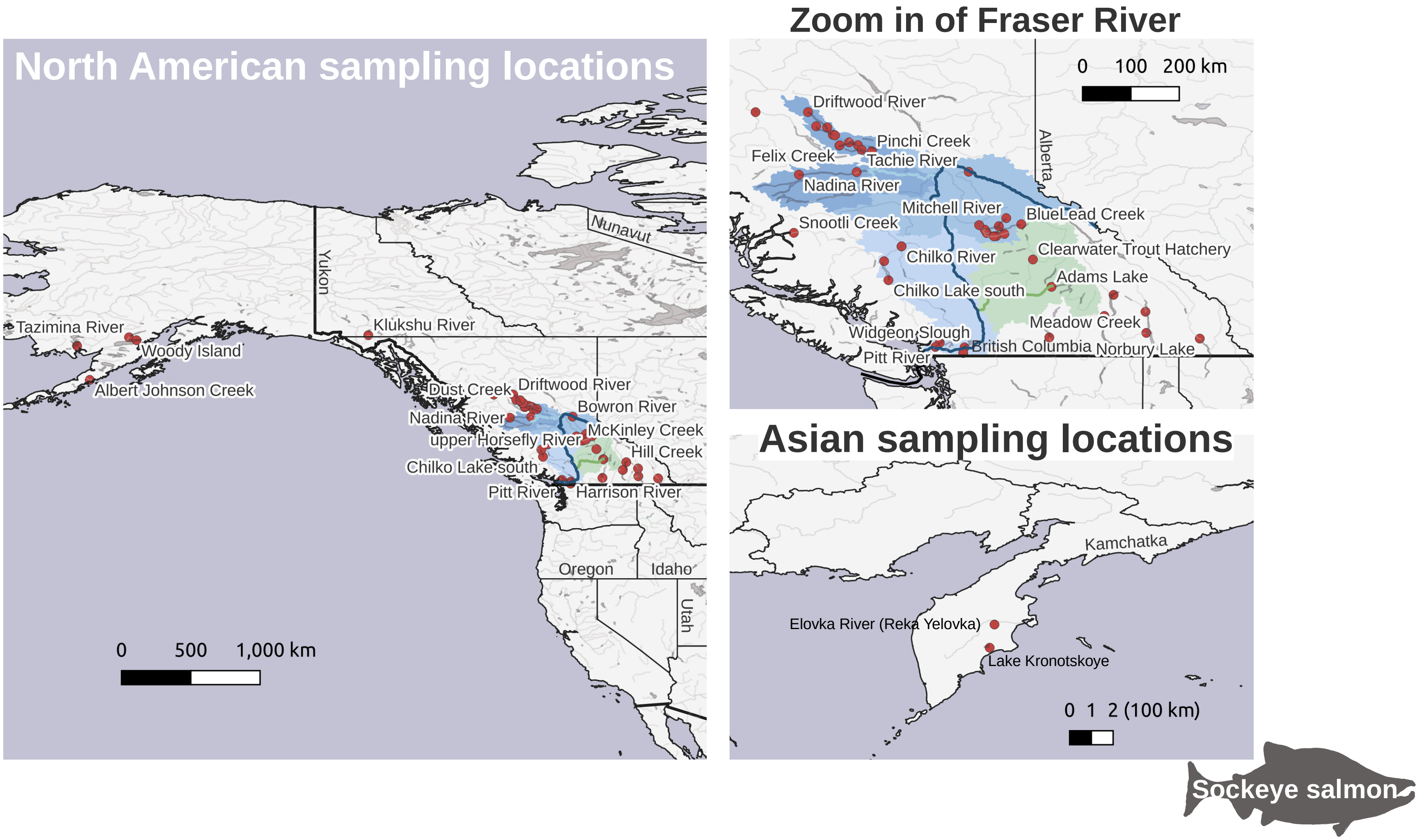
North American sampling locations
Yukon
Nunavut
Tazimina River
Woody Island
Albert Johnson Creek
Klukshu River
Dust Creek
Driftwood River
Bowron River
Nadina River
McKinley Creek
upper Horsefly River
Hill Creek
Chilko Lake south
Pitt River
Harrison River
Oregon
Idaho
Utah
0 500 1,000 km
Zoom in of Fraser River
0 100 200 km
Alberta
Driftwood River
Pinchi Creek
Felix Creek
Tachie River
Nadina River
Mitchell River
BlueLead Creek
Snootli Creek
Chilko River
Clearwater Trout Hatchery
Adams Lake
Chilko Lake south
Meadow Creek
Widgeon Slough
British Columbia
Norbury Lake
Pitt River
Asian sampling locations
Kamchatka
Elovka River (Reka Yelovka)
Lake Kronotskoye
0 1 2 (100 km)
Sockeye salmon
