# Supplementary material for: Revealing the evolutionary history and contemporary population structure of Pacific salmon in the Fraser River through genome resequencing
Source: G3 (Bethesda). 2024 Jul 23;14(10):jkae169. doi: 10.1093/g3journal/jkae169 (PMC11457079; doi:10.1093/g3journal/jkae169)
Supplement: jkae169_Supplementary_Data [file jkae169_supplementary_data.zip › Figure_S8_G3-2024-405247.pdf]

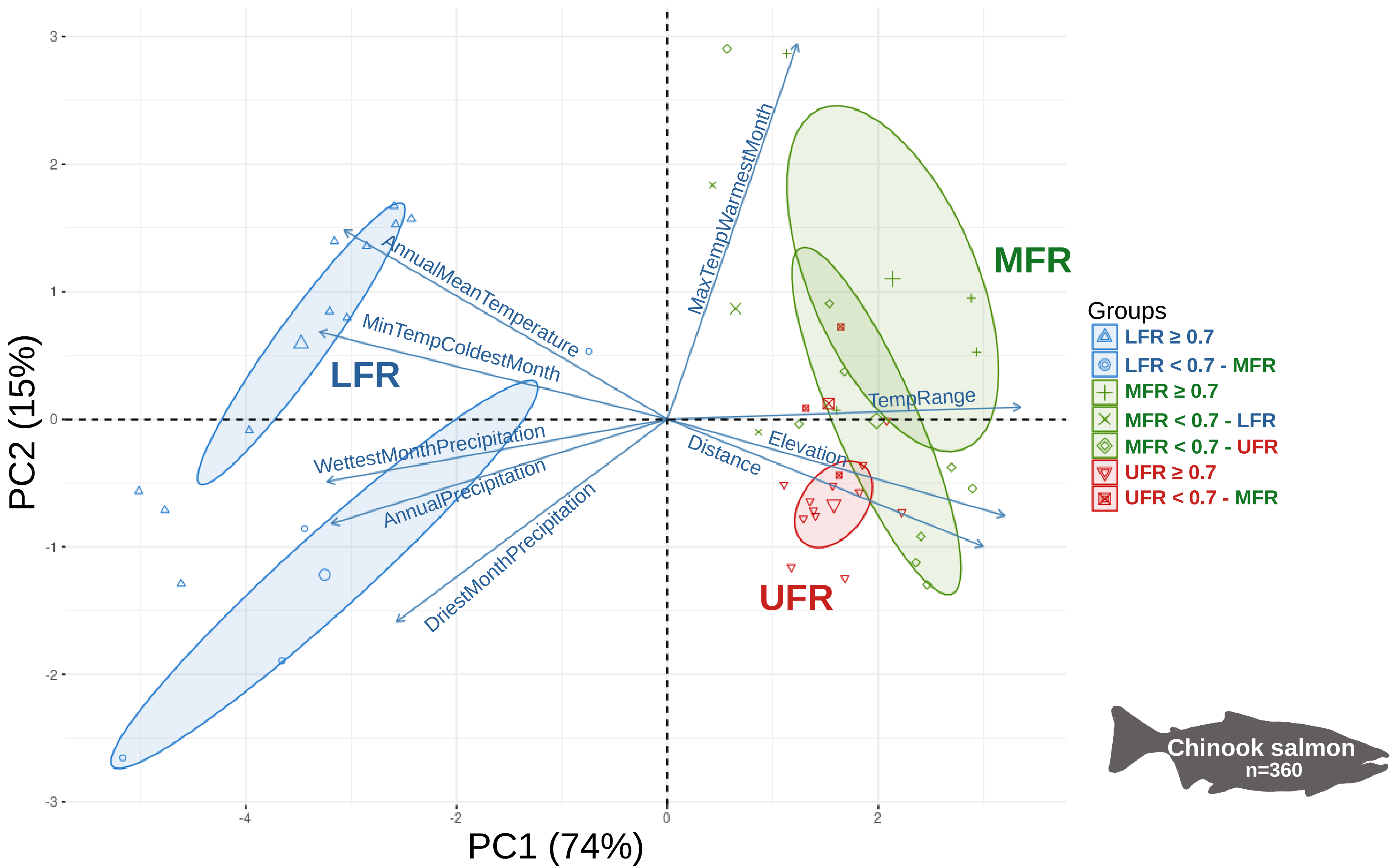

PC2 (15%)
PC1 (74%)
3
2
1
0
-1
-2
-3
-4
-2
0
2
AnnualMeanTemperature
MinTempColdestMonth
WettestMonthPrecipitation
AnnualPrecipitation
DriestMonthPrecipitation
MaxTempWarmestMonth
TempRange
Elevation
Distance
LFR
MFR
UFR
Groups
LFR ≥ 0.7
LFR < 0.7 - MFR
MFR ≥ 0.7
MFR < 0.7 - LFR
MFR < 0.7 - UFR
UFR ≥ 0.7
UFR < 0.7 - MFR
Chinook salmon
n=360

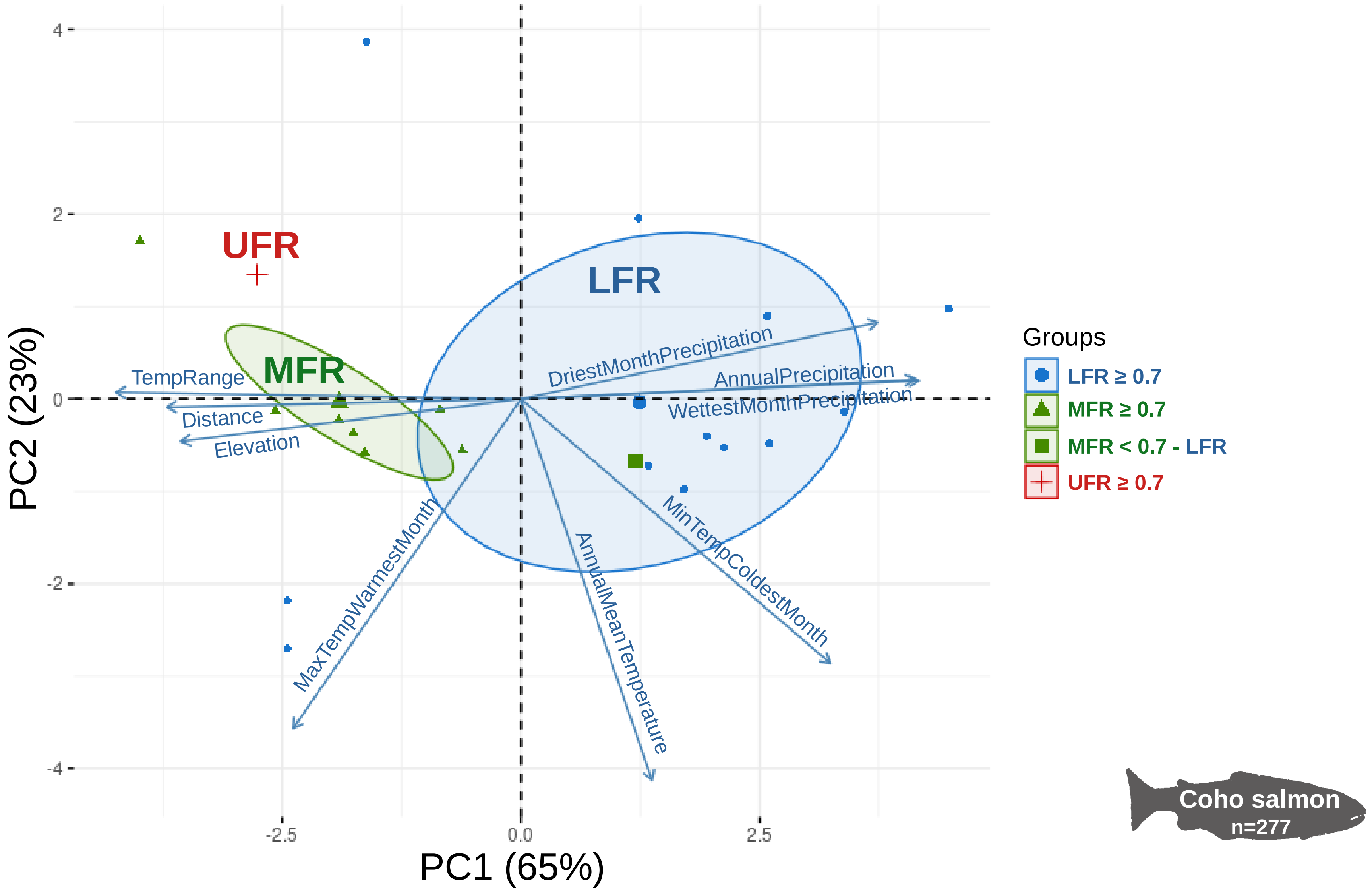

4
2
0
-2
-4
-2.5
0.0
2.5
PC2 (23%)
PC1 (65%)
UFR
LFR
MFR
TempRange
Distance
Elevation
DriestMonthPrecipitation
AnnualPrecipitation
WettestMonthPrecipitation
MaxTempWarmestMonth
AnnualMeanTemperature
MinTempColdestMonth
Groups
LFR ≥ 0.7
MFR ≥ 0.7
MFR < 0.7 - LFR
UFR ≥ 0.7
Coho salmon
n=277

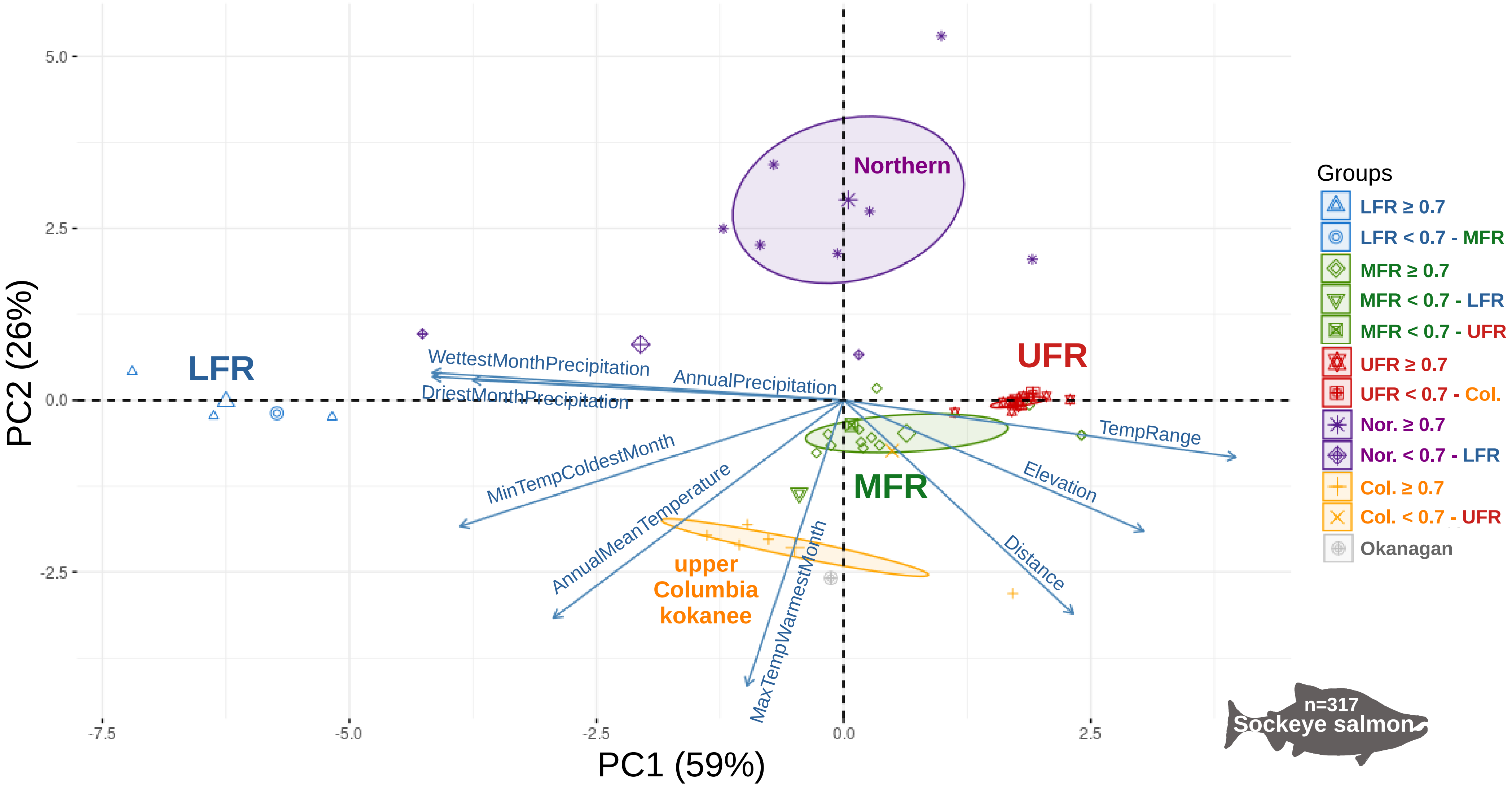

5.0
2.5
0.0
-2.5
-7.5
-5.0
-2.5
0.0
2.5
PC2 (26%)
PC1 (59%)
Northern
LFR
UFR
MFR
upper
Columbia
kokanee
WettestMonthPrecipitation
AnnualPrecipitation
DriestMonthPrecipitation
MinTempColdestMonth
AnnualMeanTemperature
MaxTempWarmestMonth
TempRange
Elevation
Distance
Groups
LFR ≥ 0.7
LFR < 0.7 - MFR
MFR ≥ 0.7
MFR < 0.7 - LFR
MFR < 0.7 - UFR
UFR ≥ 0.7
UFR < 0.7 - Col.
Nor. ≥ 0.7
Nor. < 0.7 - LFR
Col. ≥ 0.7
Col. < 0.7 - UFR
Okanagan
n=317
Sockeye salmon
