# Supplementary material for: Revealing the evolutionary history and contemporary population structure of Pacific salmon in the Fraser River through genome resequencing
Source: G3 (Bethesda). 2024 Jul 23;14(10):jkae169. doi: 10.1093/g3journal/jkae169 (PMC11457079; doi:10.1093/g3journal/jkae169)
Supplement: jkae169_Supplementary_Data [file jkae169_supplementary_data.zip › Supplemental_Material_Legends_G3-2024-405247.docx]

**Supporting Information**

**Figure S1. Timeline of glacial retreat surrounding the Fraser River.** It is thought that the Cordilleran Ice Sheet covered the Fraser River during the last glacial maximum. This map shows the retreat of glaciers from 14,000 years ago to the present glacier distribution (data from https://doi.org/10.4095/214399).

**Figure S2. Expanded maps and sampling locations.** Slide 1) Chinook, 2) coho, and 3) sockeye salmon maps. Not all sampling locations could fit in Figure 1. The maps on these slides display all sample locations. For sockeye salmon, this included North American and Asian (Russia) sampling locations.

**Figure S3. SNP quality scores along the Chinook salmon genome assembly.** Links between homeologous regions (center) were given a color/shade based on the percent identity between them (blue/dark 92 - 98%, yellow/light 88 - 92%, and green/intermediate 81 - 88%). On the next ring from the center, chromosomes were printed with tick marks every 10 Mb. SNP counts in 100 kb windows were displayed in a scatter plot on the next ring (range: 1-3580). Windows with < 500 or > 1000 SNPs were indicated with black points. Intermediate values were plotted with a lighter color (red-orange). Average quality SNP scores were plotted in 100 kb windows on the outer ring (range: 32-423,074). Windows with average quality scores below 20,000 were indicated with black points.

**Figure S4. Relationship of average SNP coverage and total length of runs of homozygosity.** Scatter plots of average SNP coverage of a) Chinook, b) coho, and c) sockeye salmon relative to the total length of runs of homozygosity (kb) for each sample. The line was plotted using the loess function (local regression) in ggplot.

**Figure S5. Pairwise relatedness among samples.** A heatmap of pairwise Ritland (Ritland 1996) relatedness scores among all samples (individual x individual) of a) Chinook, b) coho, and c) sockeye salmon. The samples labeled Illumina were sequenced with Illumina technology in a previous project. All of the other samples were sequenced with MGI technology. The relatedness scale was split (by shading) at 0.00, 0.06 (first-cousin), 0.13 (half-sib), 0.15, and 0.25 (full-sib) to highlight expected relationships (Ritland 1996). It was also split to highlight differences among groups (i.e., 0.00), and to highlight the relatedness threshold that was used in different analyses from this study (i.e., 0.15). Most of the Chinook and coho salmon samples from the previous studies had high relatedness values. These samples came mostly from coastal populations making it difficult to distinguish if the high relatedness values were the result of technical influences (e.g., SNP coverage) or influences from geography and population structure. The Thompson River (labeled) was one of the Fraser River tributaries with samples sequenced in the previous studies. These samples had low relatedness values compared to the other samples from the previous studies, suggesting an explanation related to geography rather than technical artifacts. d) Box plots of relatedness scores of pairwise values among all samples, the samples from the previous study only (Illumina), and the current study only (MGI). Box plots with the same letter above them were significantly different using a two-tailed Welch’s t-test (*p-value* < 0.05).

**Figure S6. Admixture analyses with geographic context.** a) Stacked bar plot of admixture ancestry values, with each sample represented as a column for slide 1) Chinook, 2) coho, and 3) sockeye salmon. Samples from locations with average ancestry values ≥ 0.7 were underlined with the corresponding admixture group. The 0.7 ancestry value was used as a threshold for assignment for some analyses in this study. b) Average admixture ancestry values were plotted for each sample location in QGIS using the inverse distance weighted interpolation method. The darker the sampling location, the greater the ancestry assignment to the specified admixture group. A quantile scale with 4 categories was used to better define the admixture groups visually. Some coho and sockeye salmon sample locations were outside the bounds of these maps.

**Figure S7. PCA using different SNP filtering criteria.** PCA of a) Chinook, b) coho, and c) sockeye salmon. The first column has PCA with all samples, the second column has PCA with only one individual from pairs with high relatedness scores (≥ 0.15), and the third column has PCA of only samples with high SNP coverage (>15x coverage). The different admixture groups (average ancestry values ≥ 0.7) are highlighted.

**Figure S8. PCA of sampling locations based on environmental factors.** Biplot of slide 1) Chinook, 2) coho, and 3) sockeye salmon sampling sites (points) based on environmental variables (arrows) from WorldClim version 2.1, elevation, and distance to the ocean. Sampling sites with an average ancestry assignment of less than 0.7 were assigned to intermediate genetic groups with the highest fraction named first (e.g., if a site had average ancestry values of 0.6 MFR and 0.4 LFR, it was assigned MFR < 0.7 - LFR). Okanagan sockeye salmon were an exception as they had high ancestry values for multiple genetic groups. Each sampling location is represented by a small point (unless there was only one site, in which case it has a larger symbol), larger points show the middle of ellipses (if there were enough points for the ellipse to be plotted), and ellipses were drawn at the 0.4 level.

**Figure S9. Estimates of historical effective population size for different salmon species and groups.** Slide 1) Effective population size for all species and sampling sites. Some Chinook salmon sampling locations are off the graph. These sites may have recent admixture, which can negatively influence these estimates. These locations were removed from all other figures. Slides 2-3) Fraser River admixture group comparisons of each species. Slide 4) Effective population size estimations of different groups of Chinook salmon. Sampling sites with an average ancestry assignment of less than 0.7 were assigned to intermediate admixture groups with the highest fraction named first (e.g., if a site had average ancestry values of 0.6 MFR and 0.4 LFR, it was assigned MFR < 0.7 - LFR). For the LFR, it was specified if the sites were on the Fraser River or not. Slide 5) Effective population size estimations of different groups of coho salmon. Slides 6-7) Effective population size estimations of different groups of sockeye salmon. The Okanagan was difficult to categorize into an admixture group and so was visualized separately.

**Figure S10. Total length of runs of homozygosity for each sample location.** Box plots of total runs of homozygosity (kb) for all individuals from each sample location of slide 1) Chinook, 2) coho, and 3) sockeye salmon. Locations were highlighted by admixture group (locations with average ancestry values < 0.7 were represented by mixes of admixture groups with the largest contributor first). The x-axis represents the sampling site (see File S1 for full name) and the y-axis represents the total length of runs of homozygosity within each genome.

**Figure S11. Number of polymorphic loci of each sample location.** a) The number of polymorphic loci per location is shown on the top for each species. This value represents the number of SNPs that were variant at a specific location. The number of samples per location is shown at the bottom. b) Scatter plot of the number of samples per location and the number of polymorphic loci identified from those locations. The line was added using the gam method in ggplot. c) A box plot of the number of polymorphic loci based on the admixture group. Locations with average admixture group ancestry values ≥ 0.7 only have one label (i.e., lower Fraser River – LFR, mid Fraser River – MFR, and upper Fraser River – UFR). Those with < 0.7 have two labels with the first having the largest ancestry value. Only locations with at least four samples were used for this analysis. d) Scatter plot of the number of polymorphic loci (x-axis) and nucleotide diversity (y-axis) for locations with at least four individuals per site. Each species has its linear regression line plotted.

**Figure S12. Extended haplotype homozygosity comparisons between LFR and MFR admixture groups.** A Manhattan plot of -log10 *p-values* from an Rsb analysis between LFR and MFR admixture groups of a) Chinook, b) coho, and c) sockeye salmon. Sockeye salmon chromosomes were based on a draft genome assembly submitted to the NCBI (now GCA_034236695.1). Significant peaks were indicated on the plots with a vertical line and a number.

**File S1. Sample summary information in a spreadsheet format.** If individuals were assigned to an admixture group with an ancestry value ≥ 0.7, only one label was used (e.g., lower Fraser River – LFR). If the value was less, it was given a label of the top two groups, with the highest assignment first (e.g., LFR-MFR). Only the Okanagan samples from sockeye salmon deviated from this pattern, and they were given their own admixture group name even though they were not a separate cluster in the analysis.

**File S2. Truth SNPs used in GATK recalibration for each species (includes a readme file).**

**File S3. Significant extended haplotype homozygosity from all comparisons among groups.**
