# Supplementary material for: Revealing the evolutionary history and contemporary population structure of Pacific salmon in the Fraser River through genome resequencing
Source: G3 (Bethesda). 2024 Jul 23;14(10):jkae169. doi: 10.1093/g3journal/jkae169 (PMC11457079; doi:10.1093/g3journal/jkae169)
Supplement: jkae169_Supplementary_Data [file jkae169_supplementary_data.zip › Table_S1_G3-2024-405247.docx]

**Table S1. SNP filtering parameters that were used for each analysis.**

| **Analyses** | **Indels, >10% missing, depth** | **MAF 0.01** | **LD Filter** | **15x coverage** | **Relatedness** |
| --- | --- | --- | --- | --- | --- |
| SMC++ | all |  |  |  |  |
| Rehh | all | all |  |  |  |
| Coverage | all | all |  |  |  |
| ROH | all | all |  |  |  |
| Relatedness | all | all | all |  |  |
| Admixture | all | all | all |  |  |
| *F_st_* | all | all |  | all |  |
| Nucleotide diversity | all | all |  | all |  |
| Polymorphic loci | all | all |  | all |  |
| PCA | all | all | all | some* | some* |

“All” in this table means that every type of this analysis used the same SNP filtering parameters. “Some” means that not all analyses of this type used the specified filtering. *See Figure S7 for all PCA, including some with the 15x SNP coverage and relatedness filters.
