# Supplementary figures and images for: Revealing the evolutionary history and contemporary population structure of Pacific salmon in the Fraser River through genome resequencing
Source: G3 (Bethesda). 2024 Jul 23;14(10):jkae169. doi: 10.1093/g3journal/jkae169 (PMC11457079; doi:10.1093/g3journal/jkae169)

# Chinook salmon

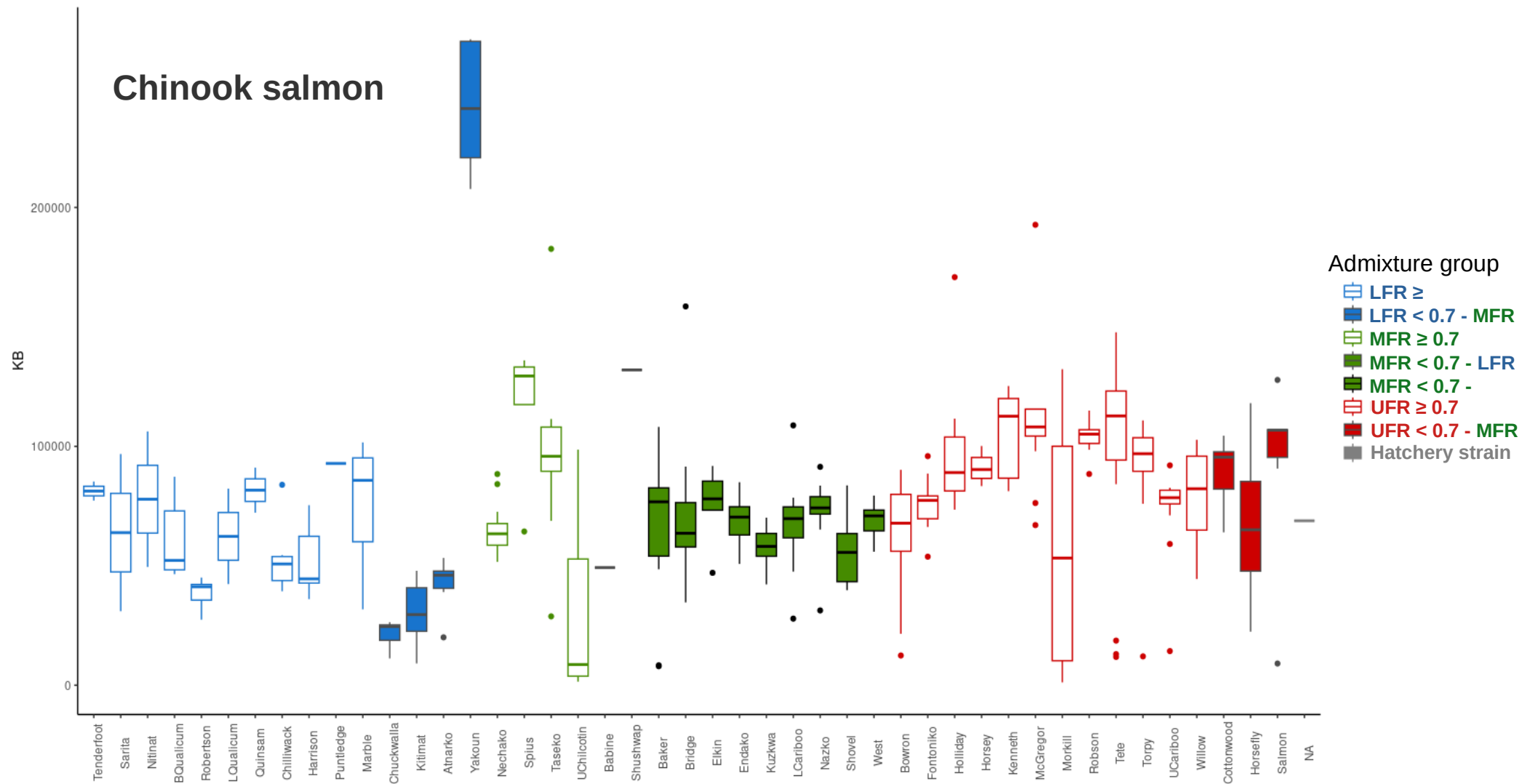

# Coho salmon

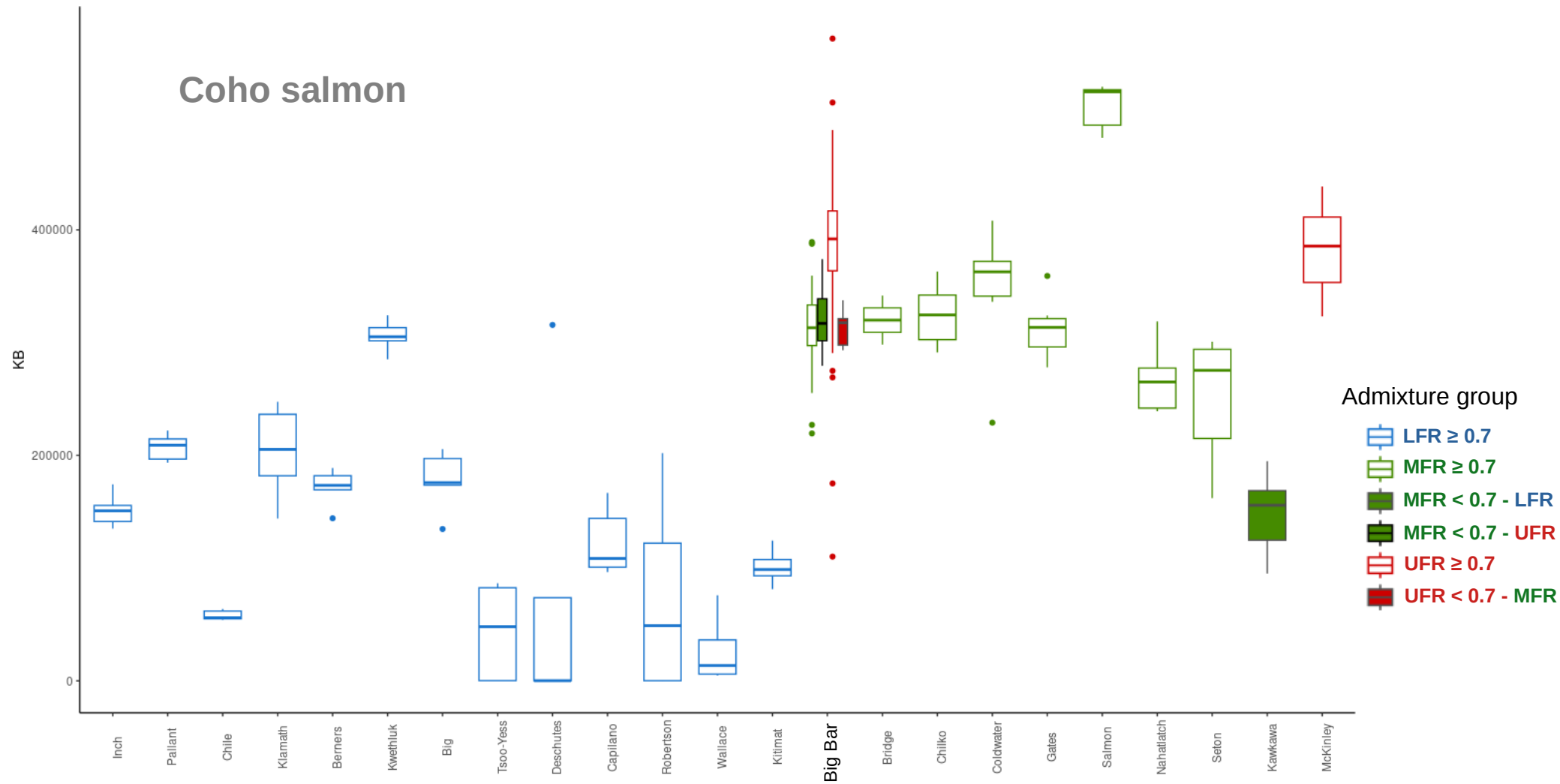

# Sockeye salmon

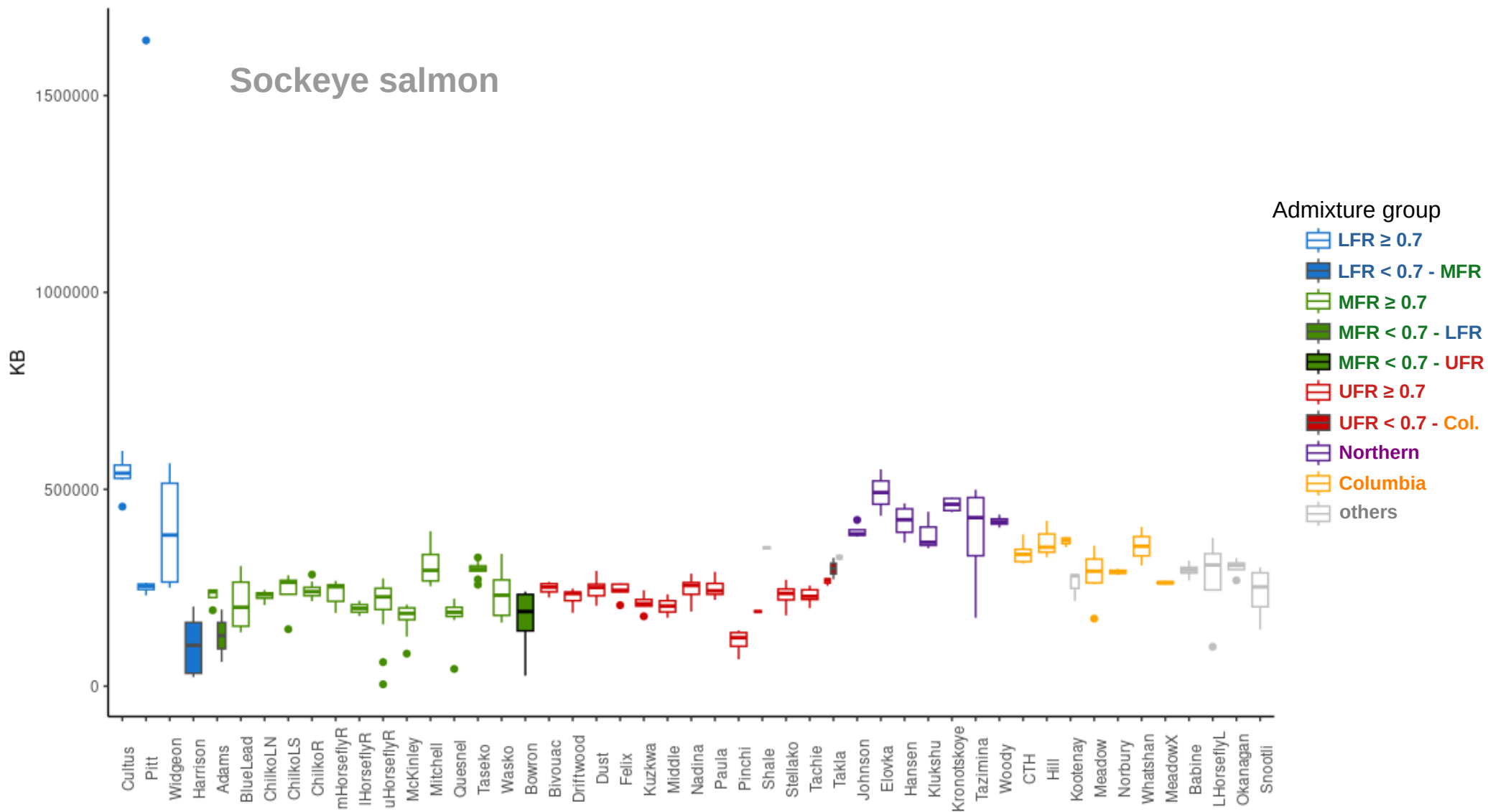

Supplement: jkae169_Supplementary_Data [file jkae169_supplementary_data.zip › Figure_S10_G3-2024-405247.pdf]

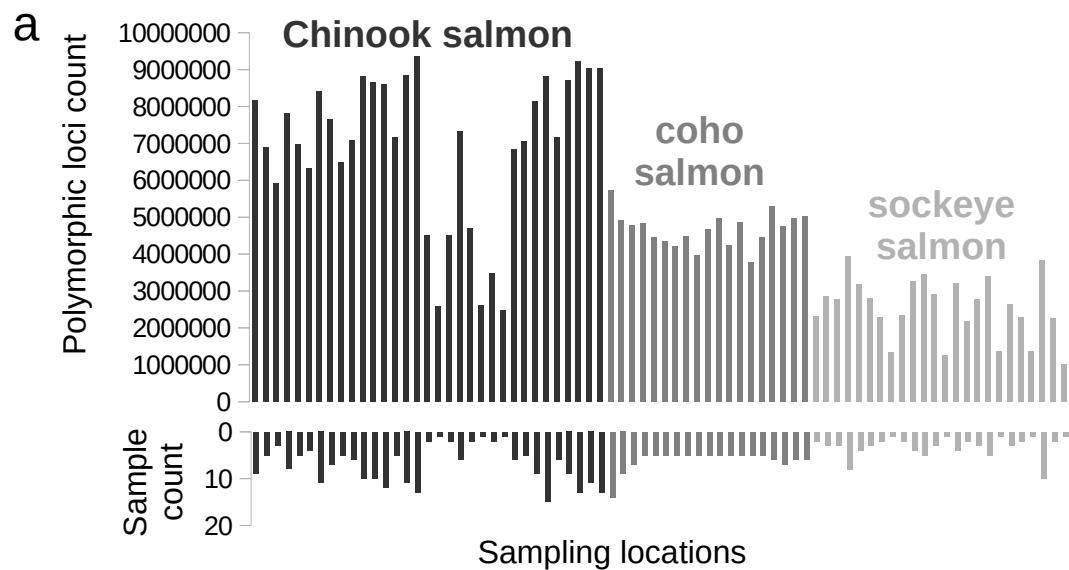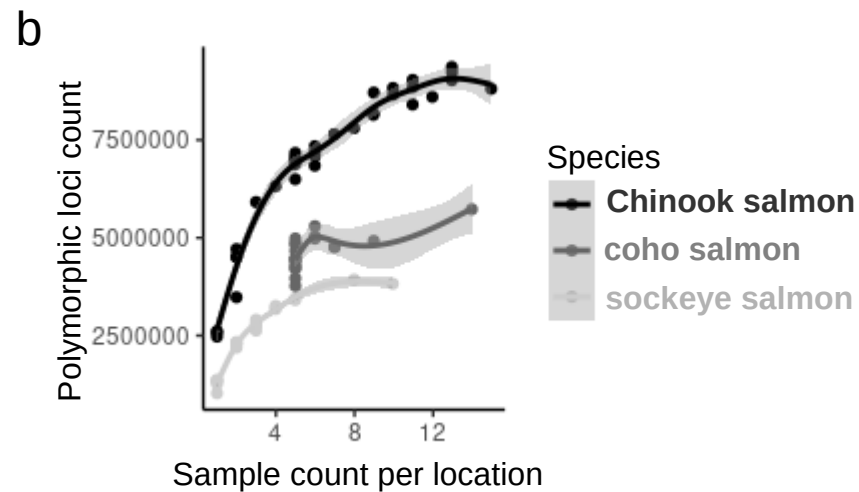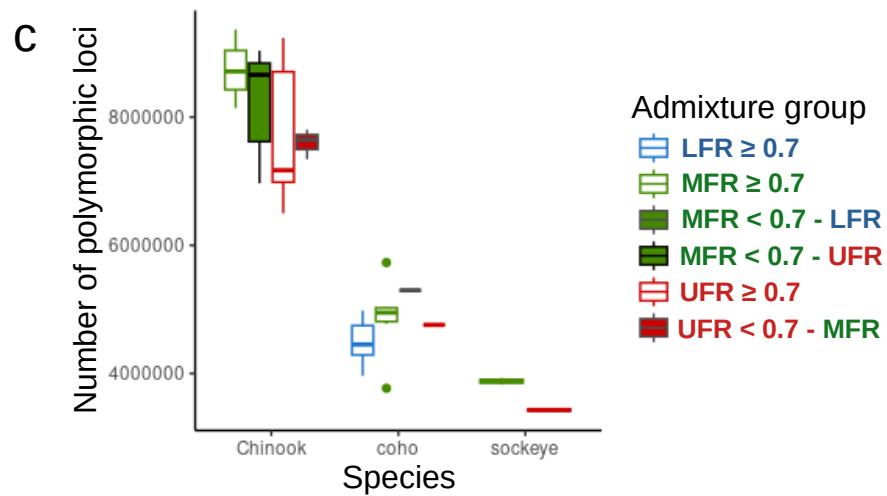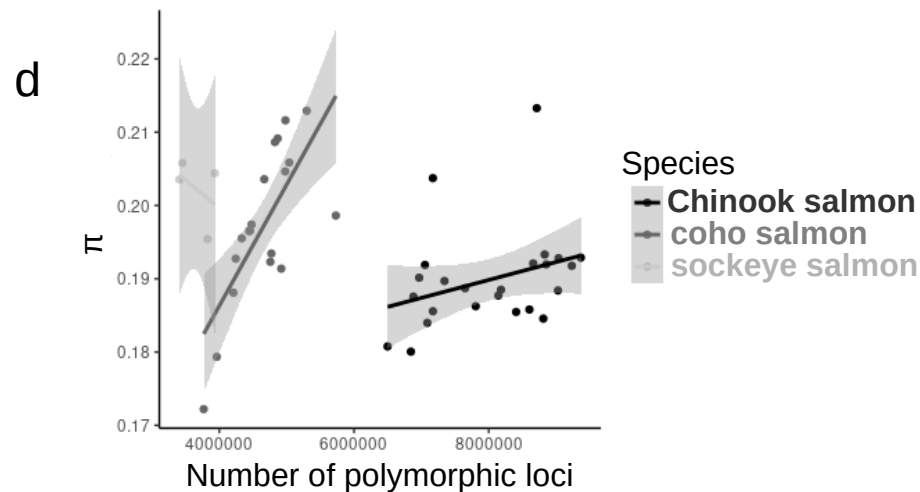

Supplement: jkae169_Supplementary_Data [file jkae169_supplementary_data.zip › Figure_S11_G3-2024-405247.pdf]

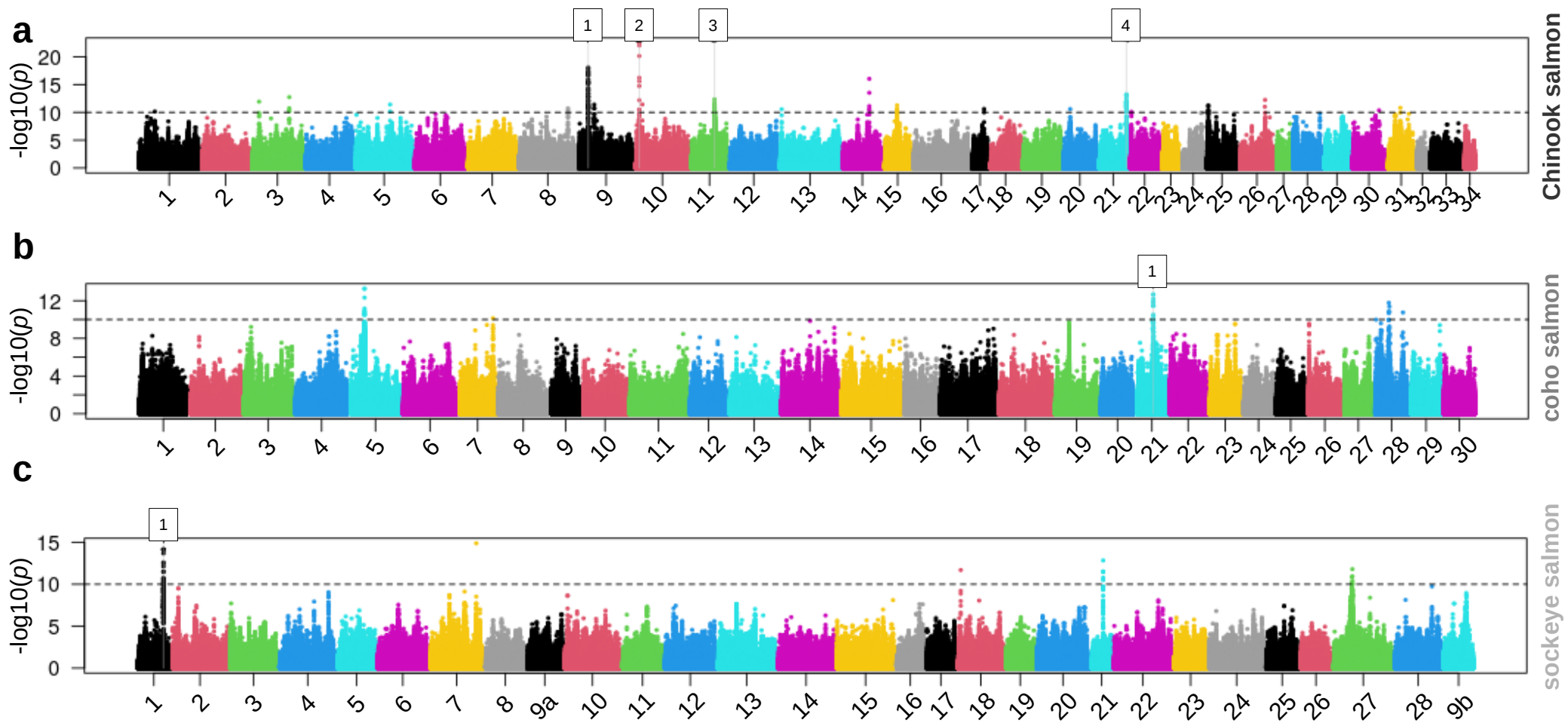

Supplement: jkae169_Supplementary_Data [file jkae169_supplementary_data.zip › Figure_S12_G3-2024-405247.pdf]

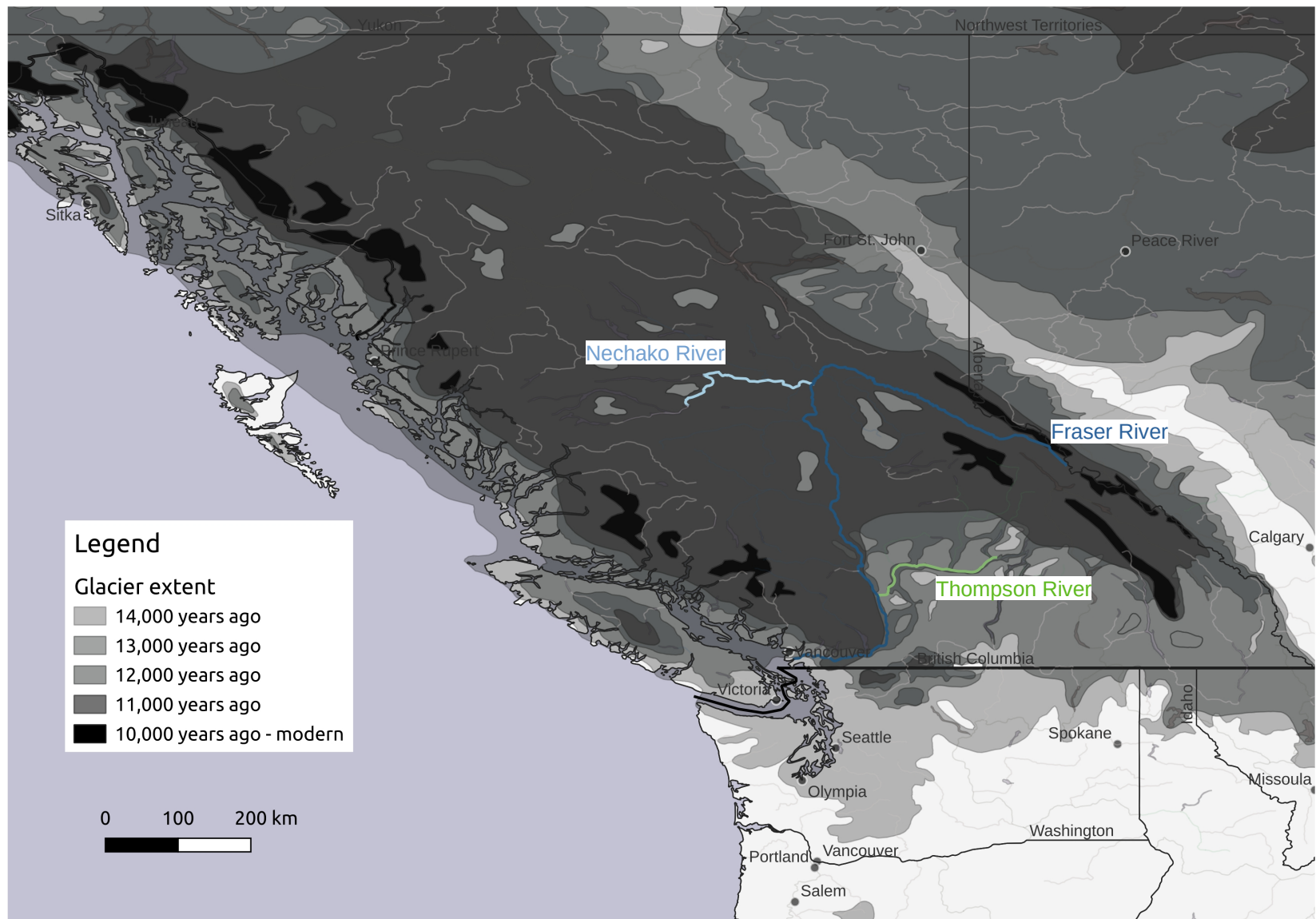

Supplement: jkae169_Supplementary_Data [file jkae169_supplementary_data.zip › Figure_S1_G3-2024-405247.pdf]

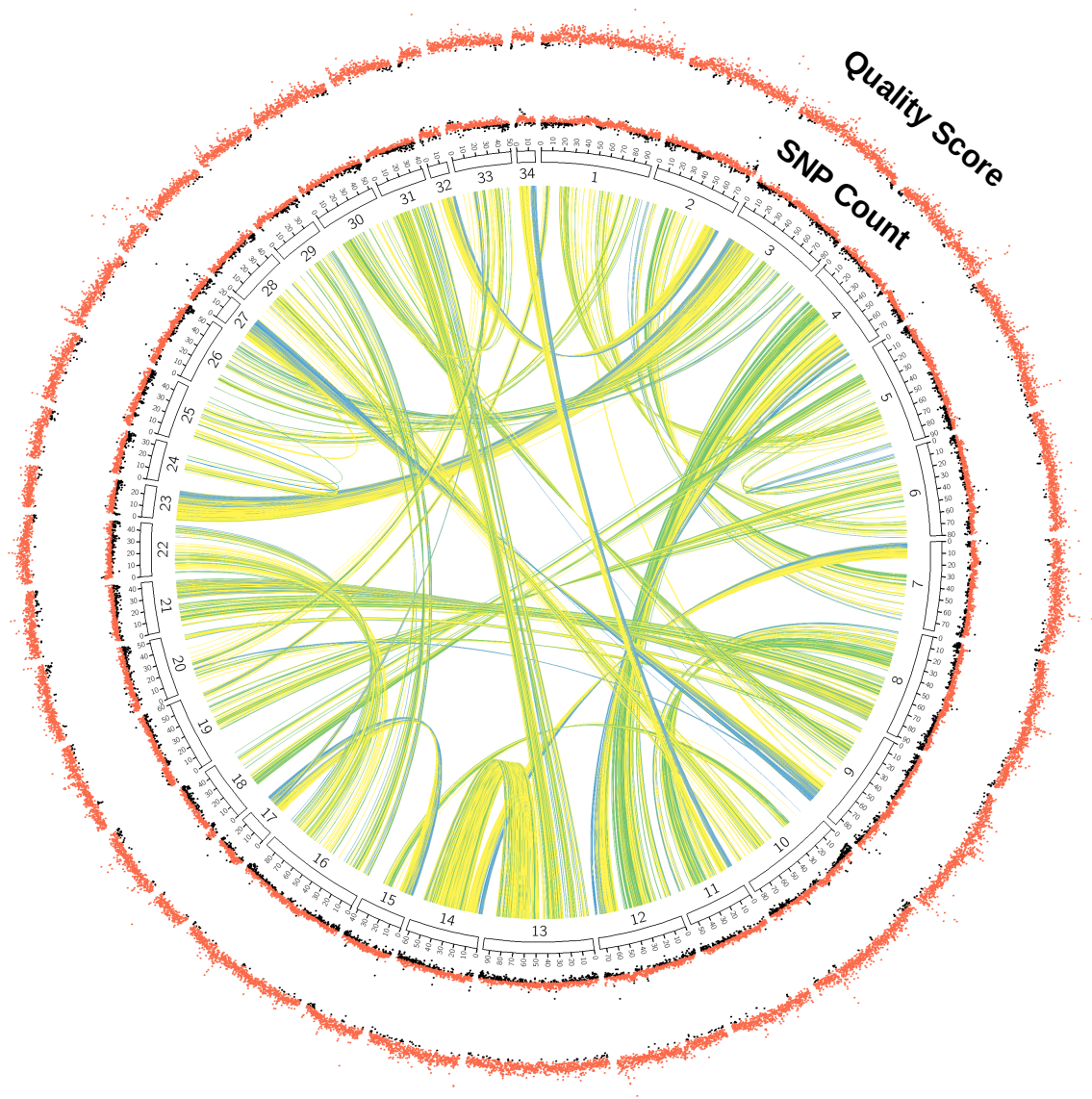

Supplement: jkae169_Supplementary_Data [file jkae169_supplementary_data.zip › Figure_S3_G3-2024-405247.pdf]

**a**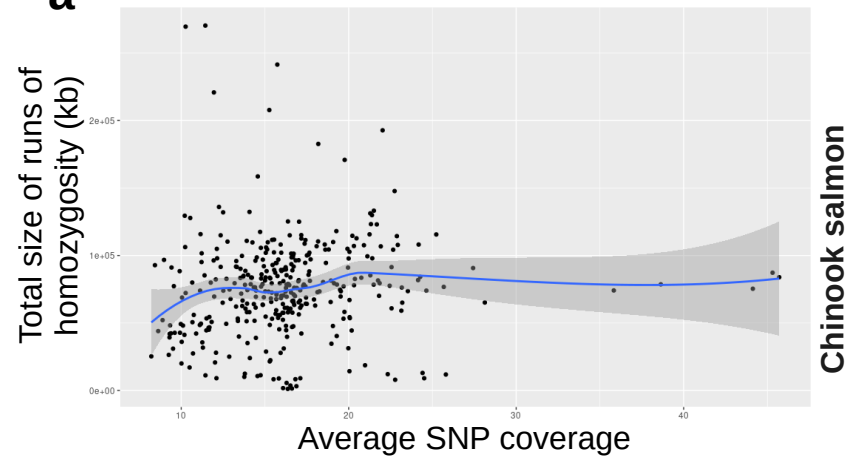**b**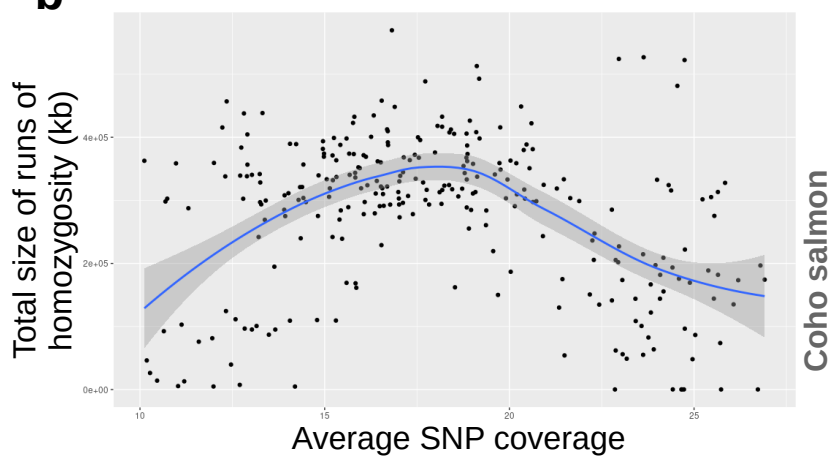**c**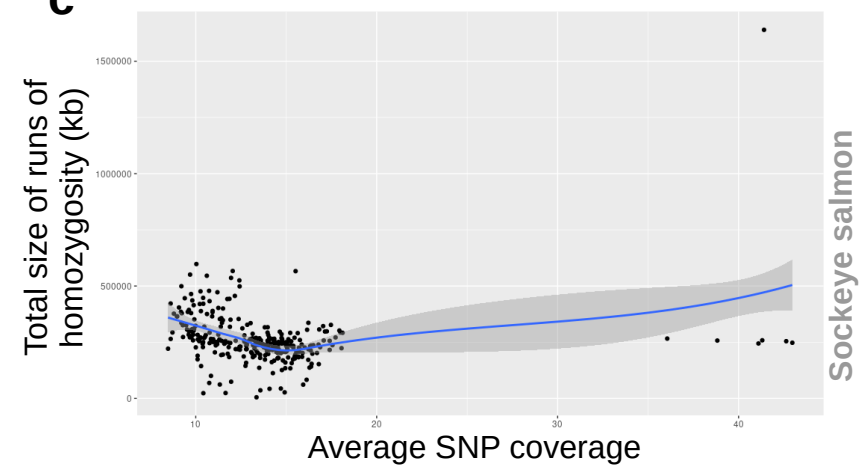

Supplement: jkae169_Supplementary_Data [file jkae169_supplementary_data.zip › Figure_S4_G3-2024-405247.pdf]

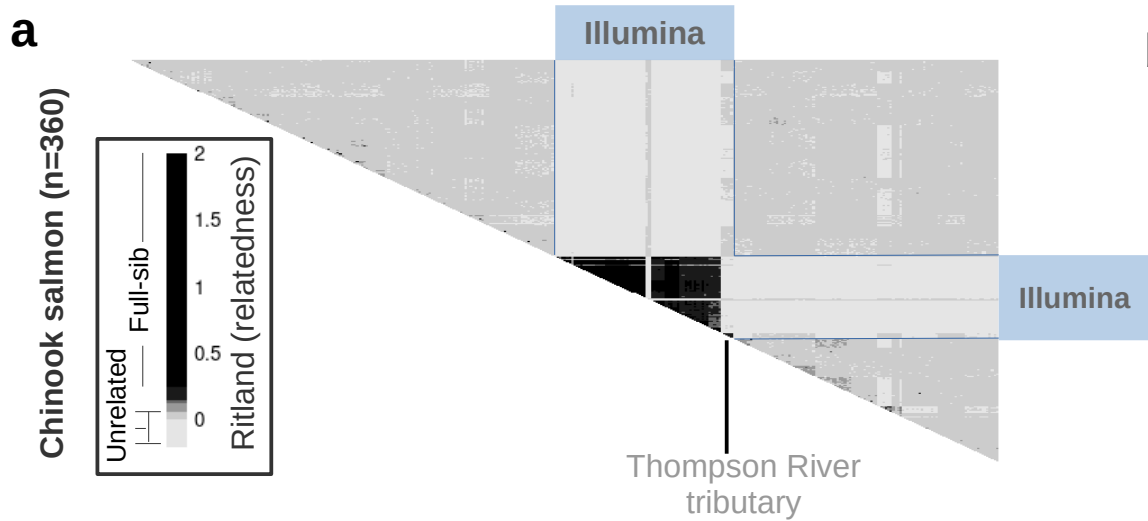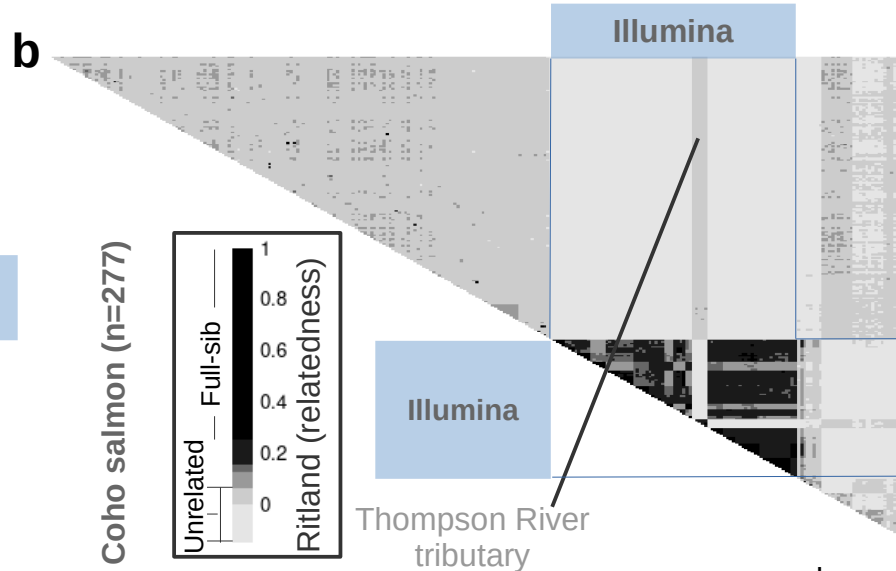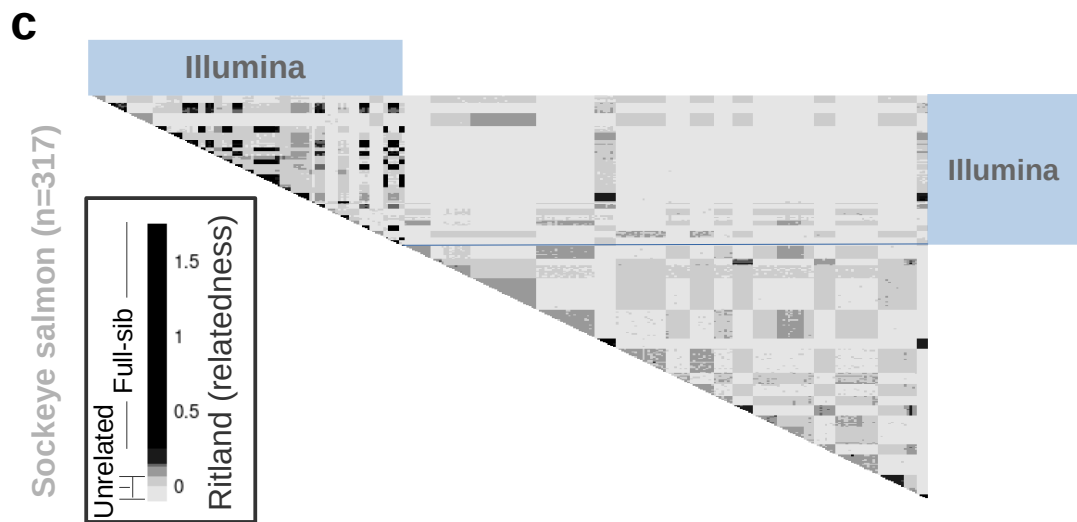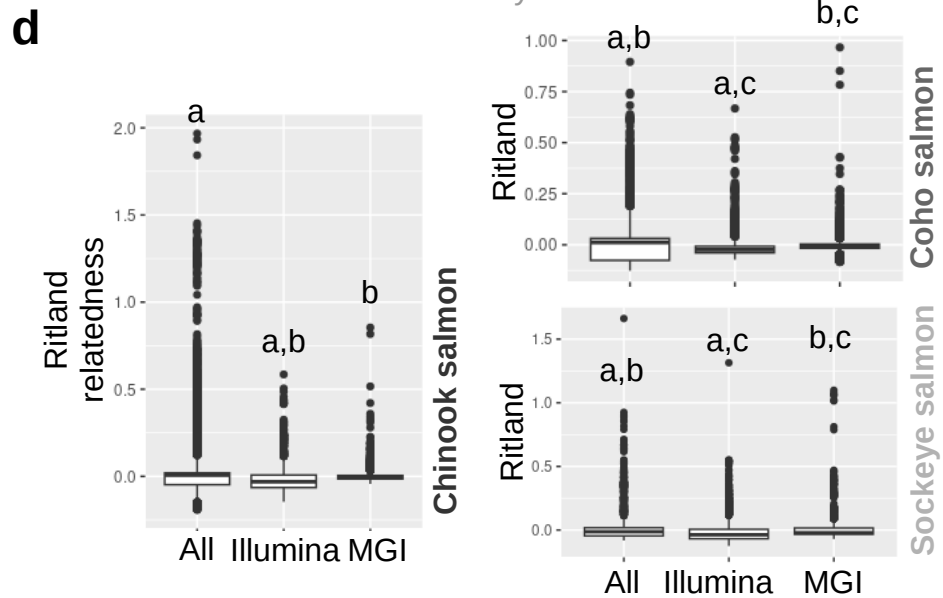

Supplement: jkae169_Supplementary_Data [file jkae169_supplementary_data.zip › Figure_S5_G3-2024-405247.pdf]

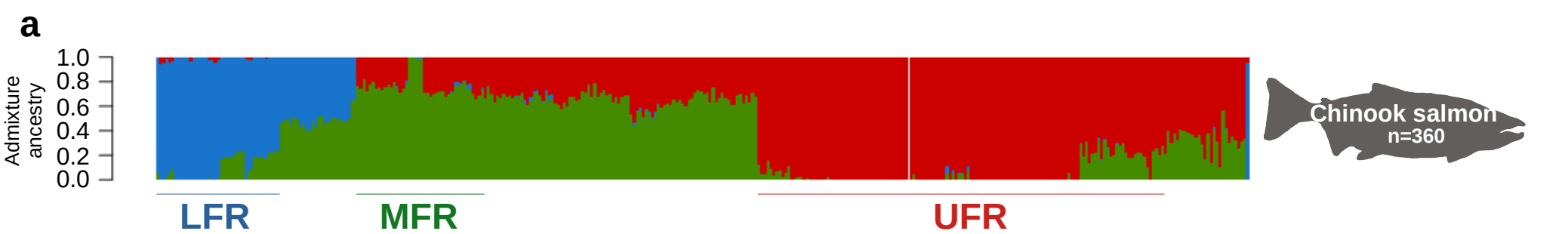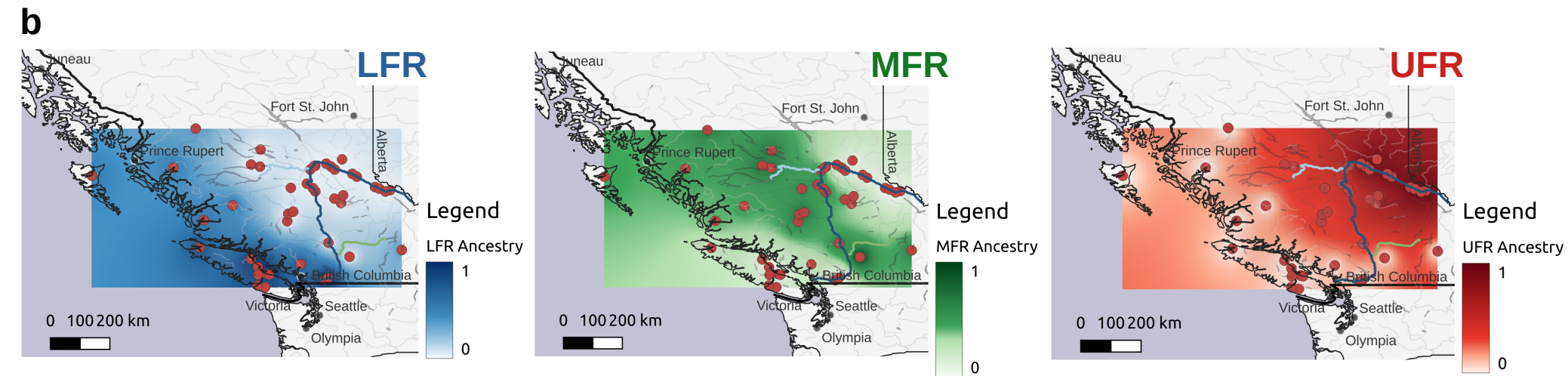

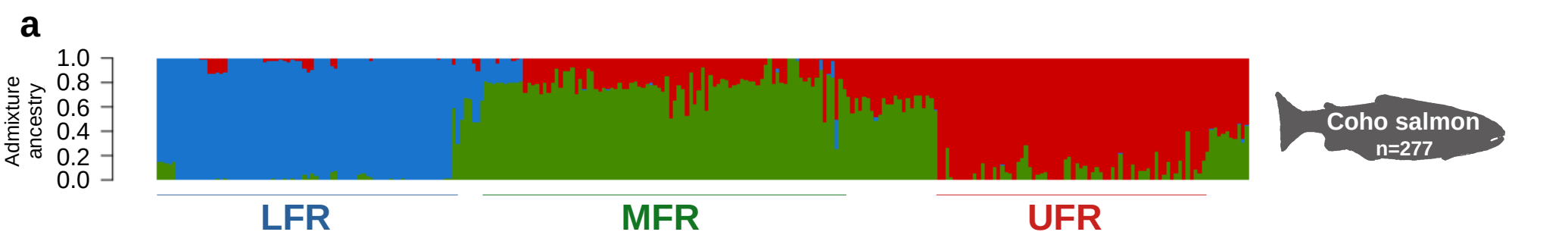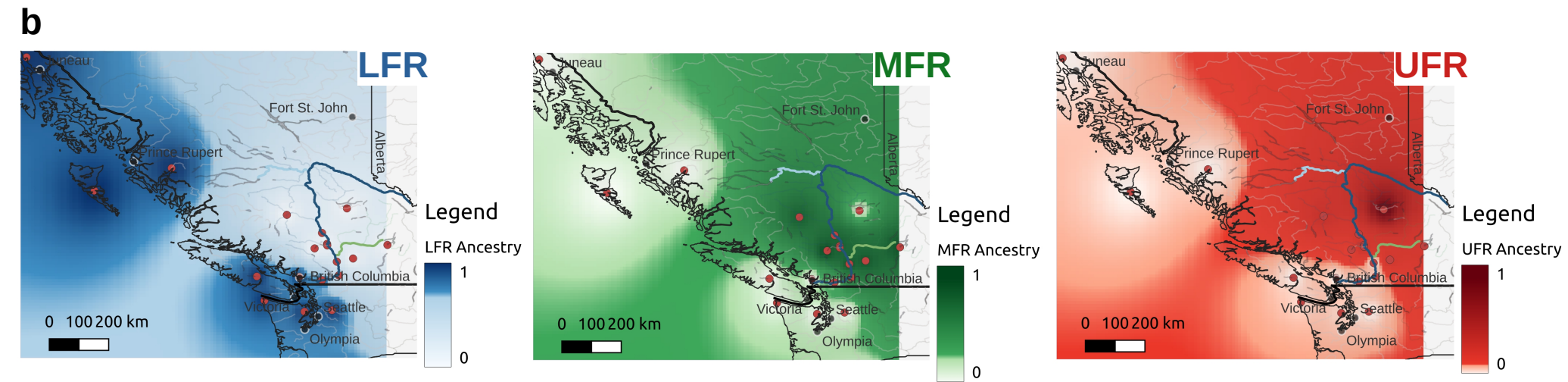

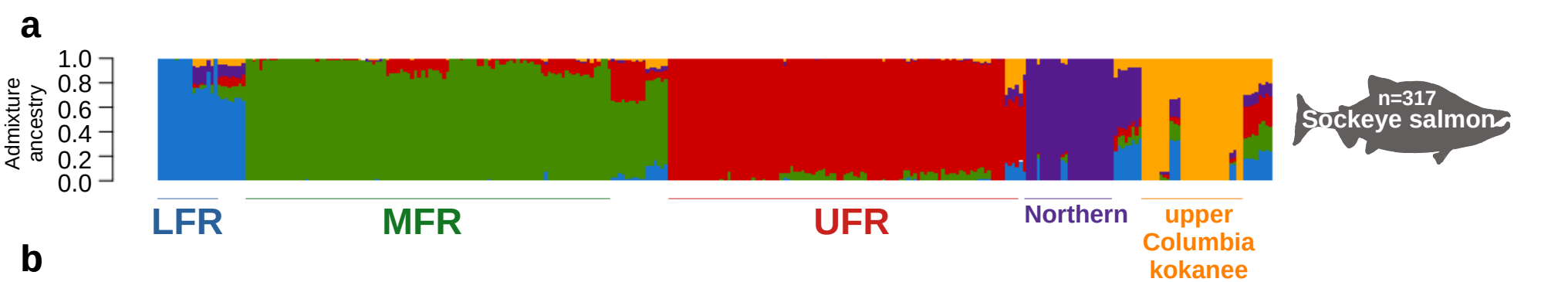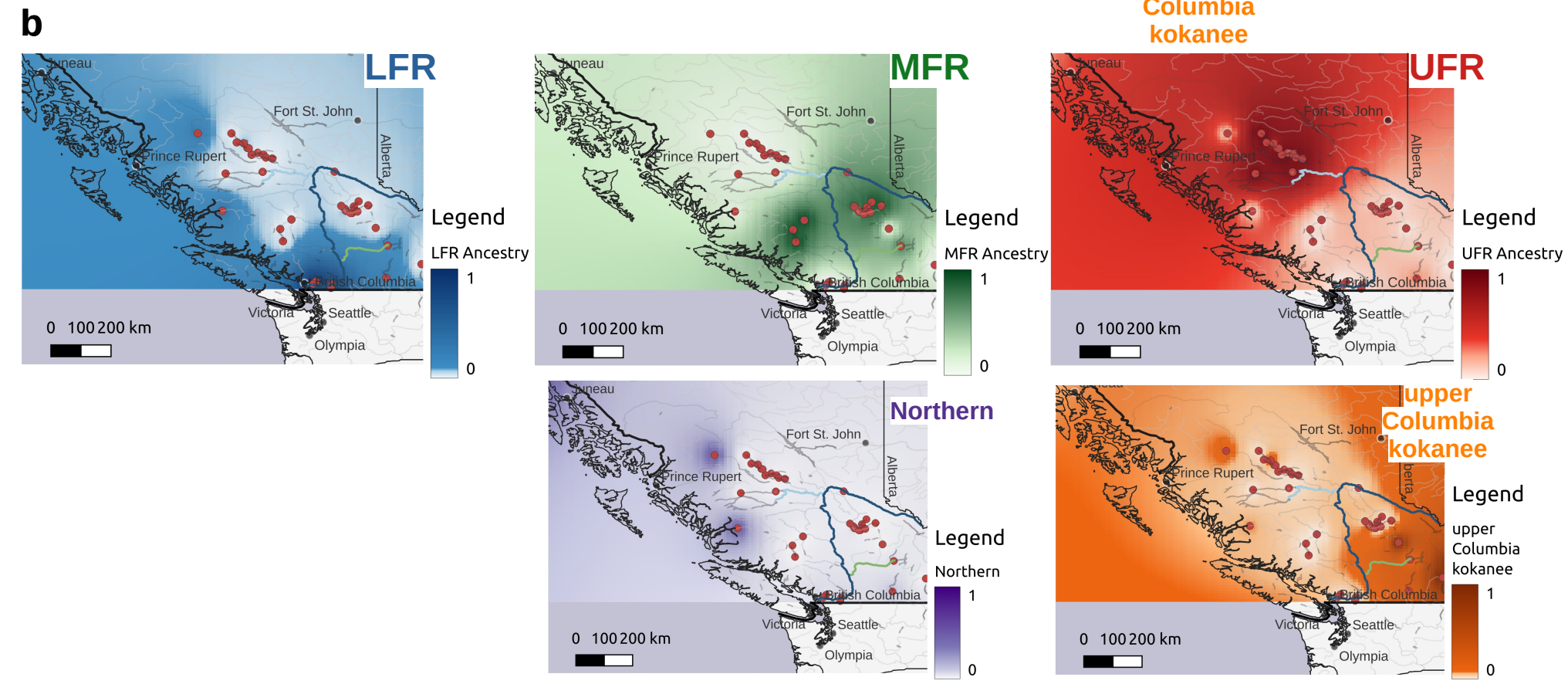

Supplement: jkae169_Supplementary_Data [file jkae169_supplementary_data.zip › Figure_S6_G3-2024-405247.pdf]

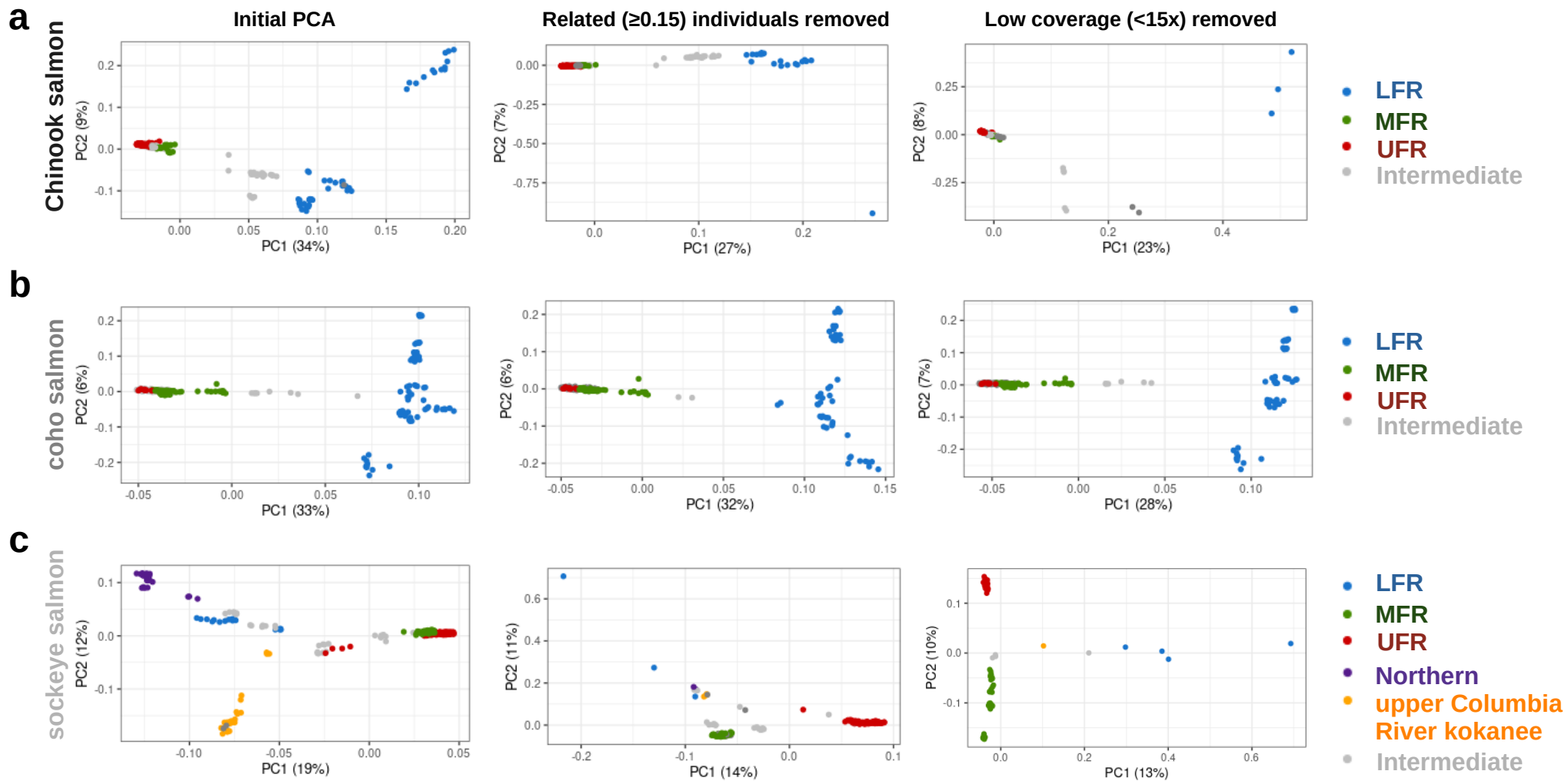

Supplement: jkae169_Supplementary_Data [file jkae169_supplementary_data.zip › Figure_S7_G3-2024-405247.pdf]

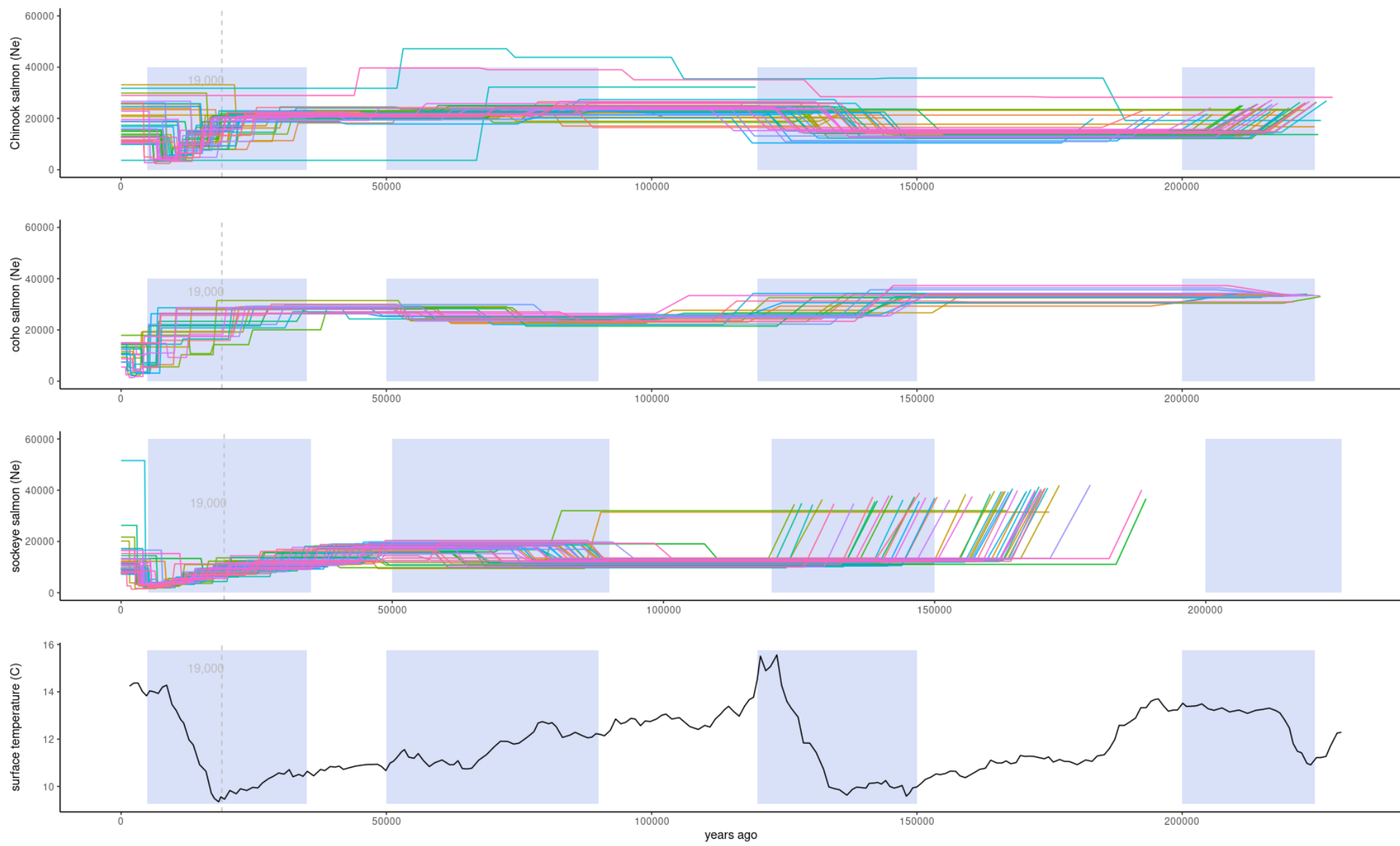

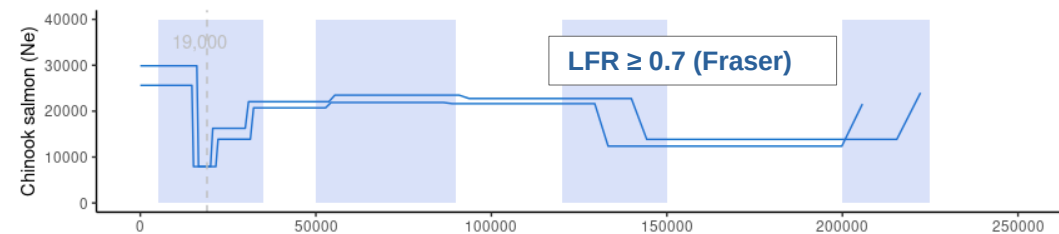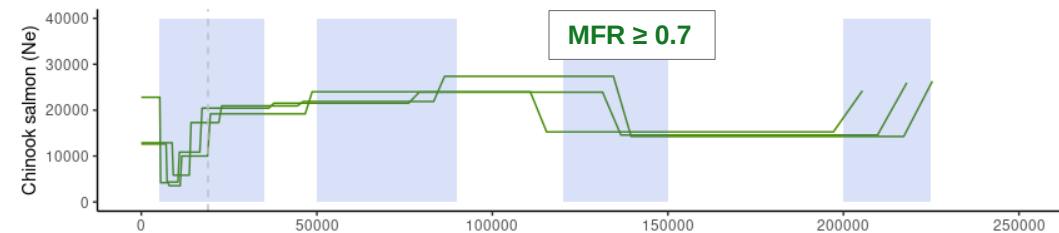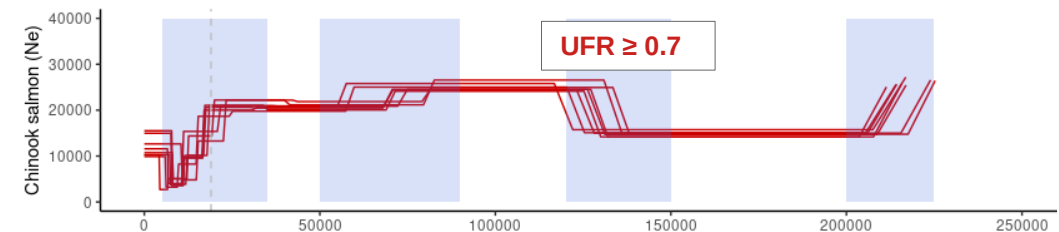

Chinook salmon

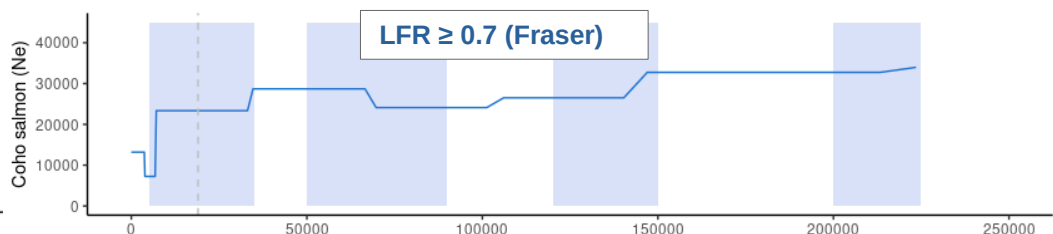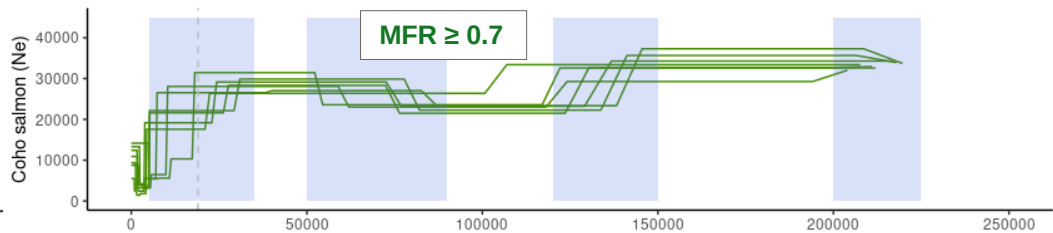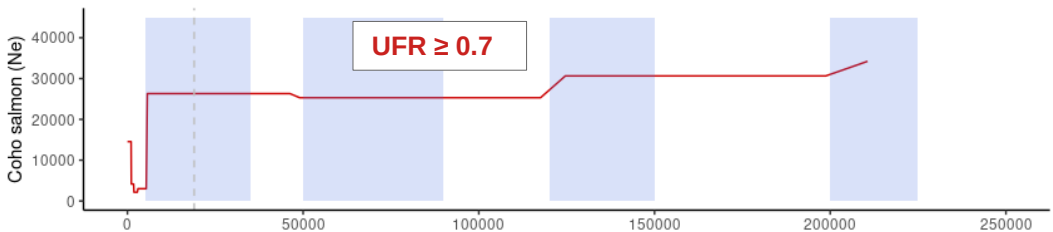

coho salmon

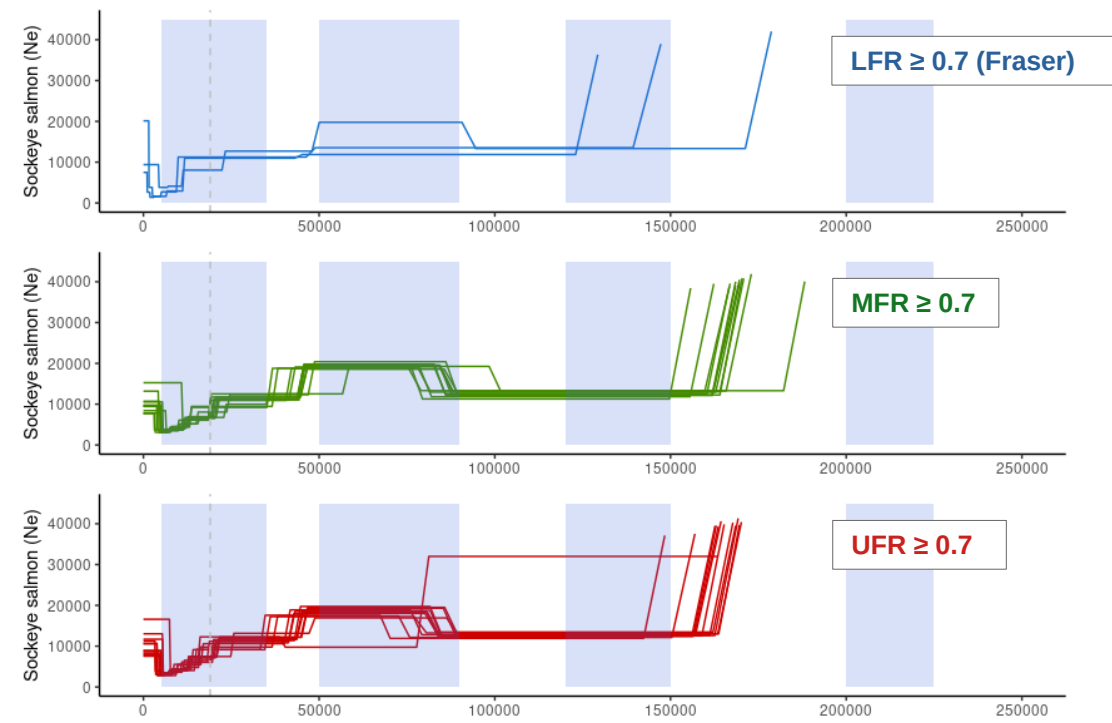

sockeye salmon

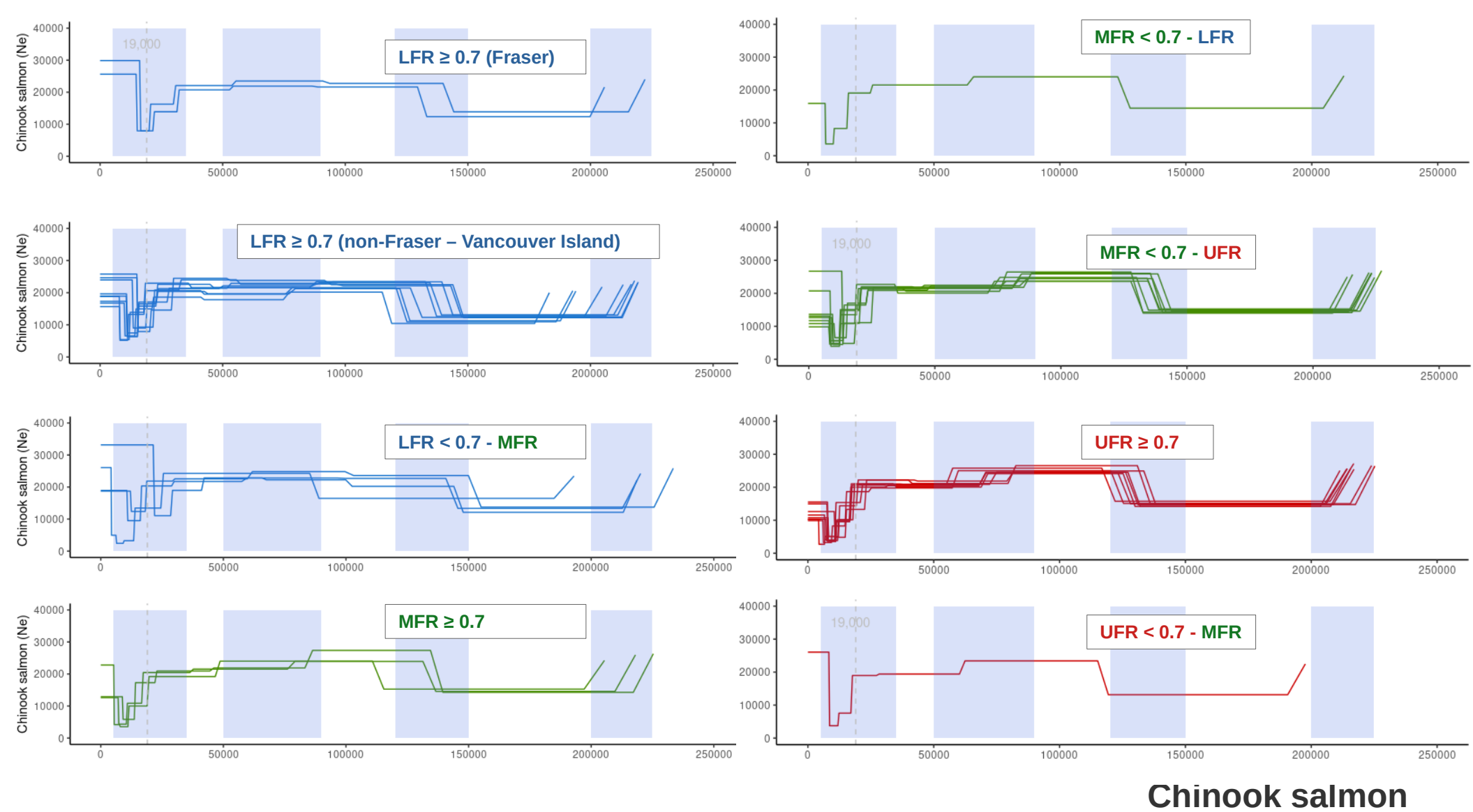

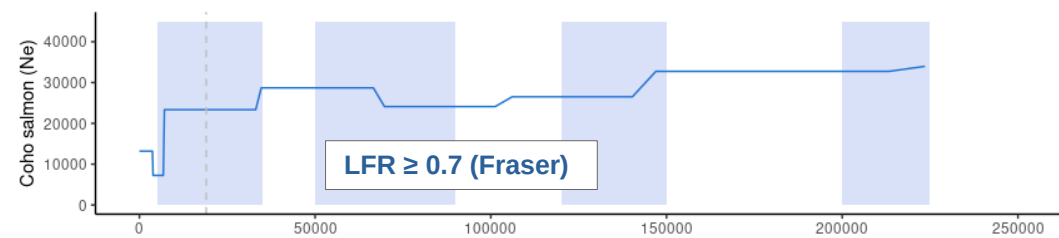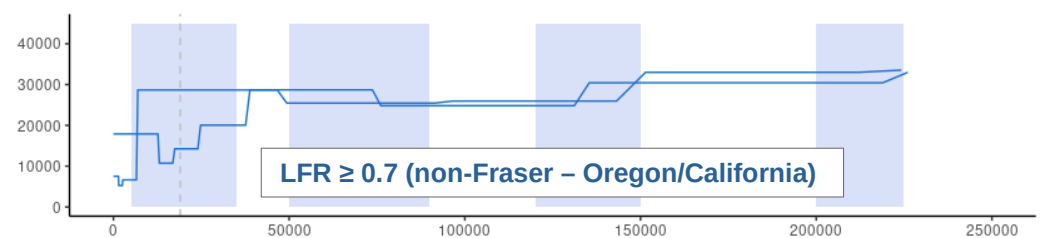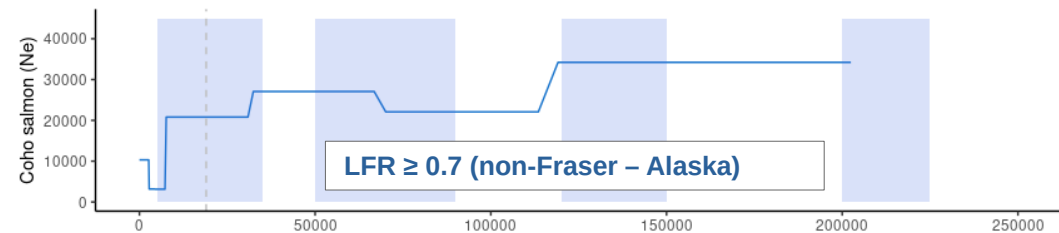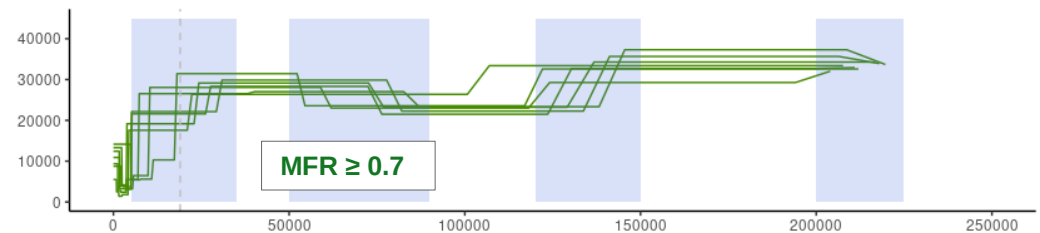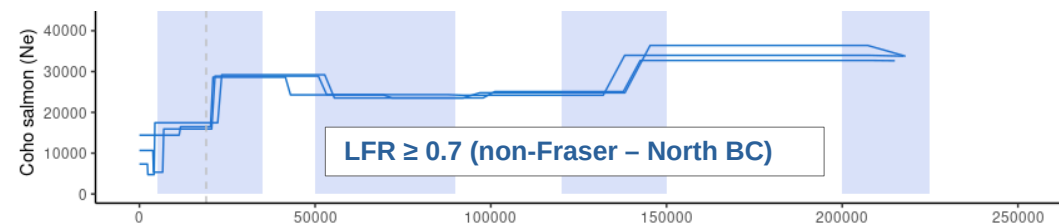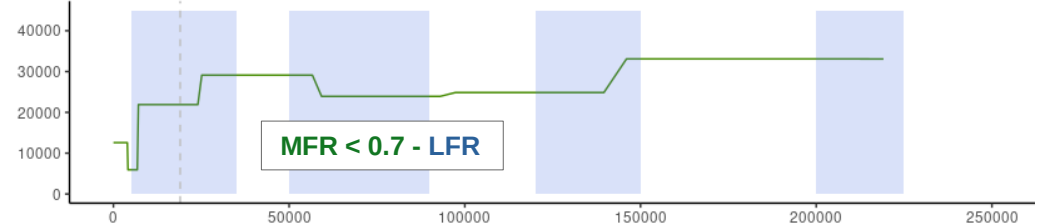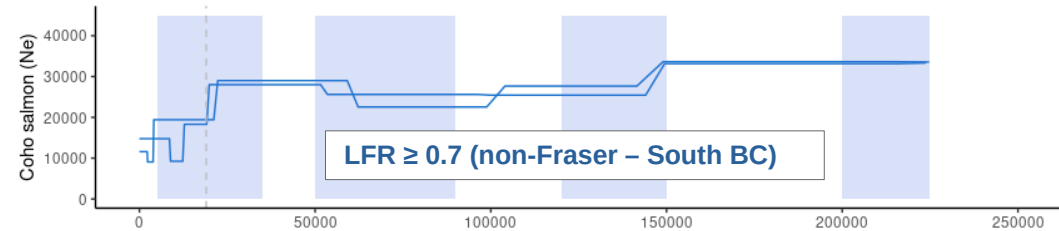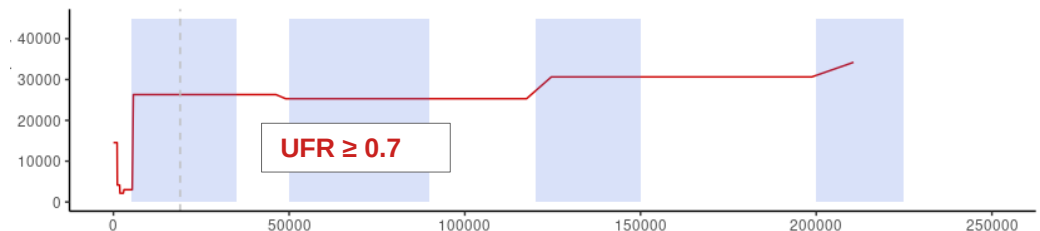

coho salmon

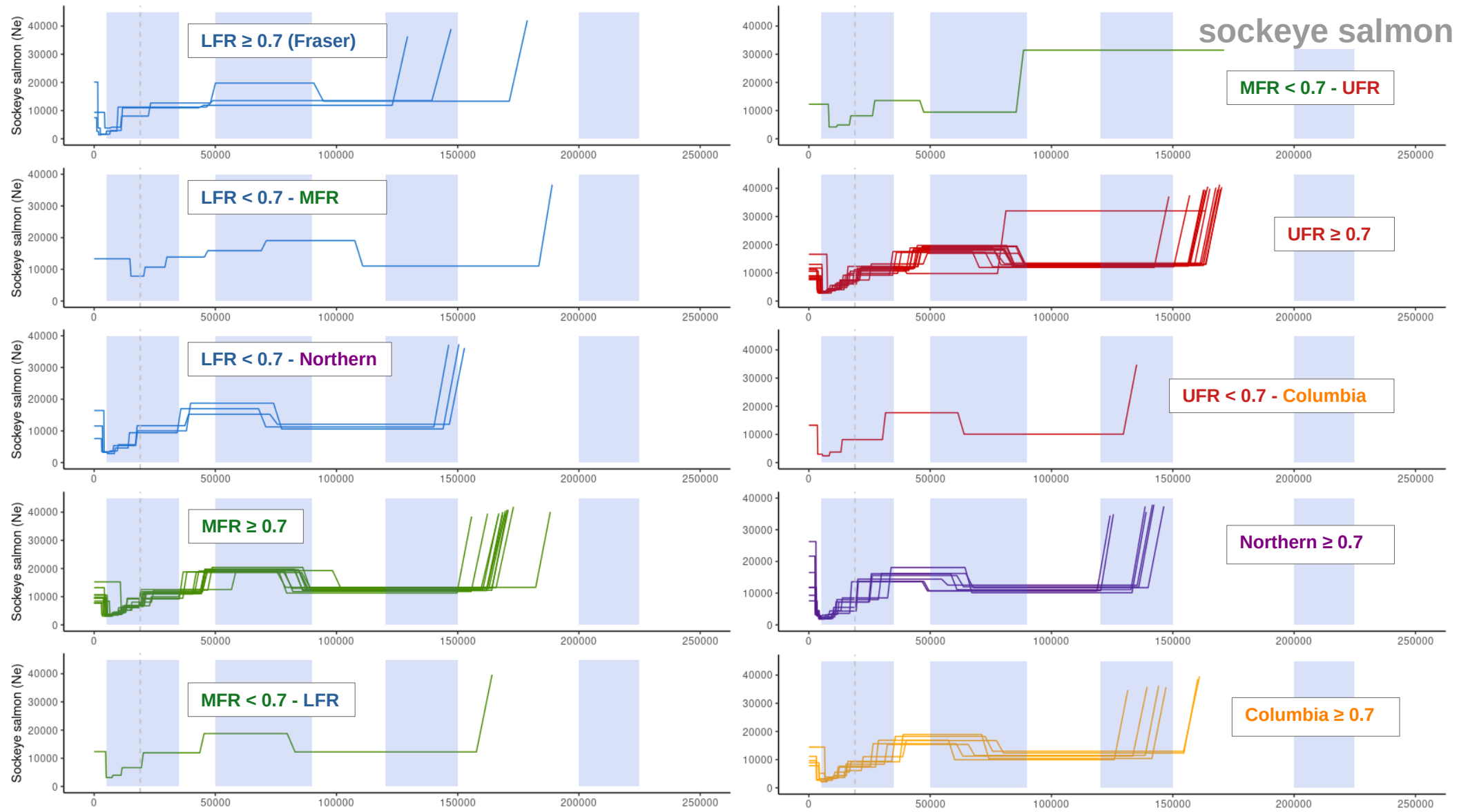

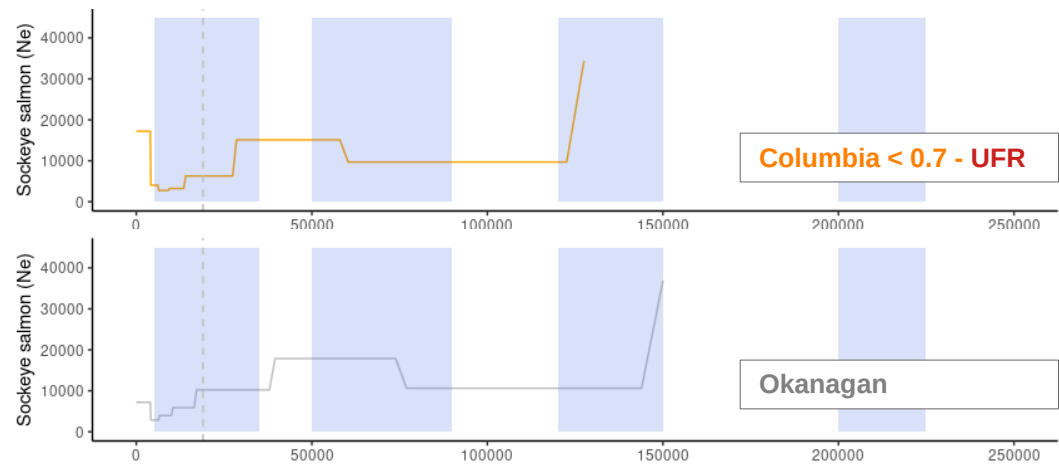

sockeye salmon

Supplement: jkae169_Supplementary_Data [file jkae169_supplementary_data.zip › Figure_S9_G3-2024-405247.pdf]
